# Structural Phase Transitions and Magnetic Characterization of Ba_2_GdNbO_6_ for Low-Temperature Magnetocaloric Refrigeration

**DOI:** 10.1021/acs.chemmater.5c01937

**Published:** 2025-10-27

**Authors:** Fiamma Berardi, Liam A. V. Nagle-Cocco, James M. A. Steele, Xiaotian Zhang, Cheng Liu, Henry E. Fischer, Siân E. Dutton

**Affiliations:** † Cavendish Laboratory, 2152 University of Cambridge, JJ Thomson Avenue, Cambridge CB3 0US, U.K.; ‡ Yusuf Hamied Department of Chemistry, University of Cambridge, Cambridge CB2 1EW, U.K.; § 56053 Institut Laue-Langevin, 71 Avenue des Martyrs, CS 20156, Grenoble cedex 9 38042, France

## Abstract

Ba_2_GdNbO_6_ has previously been reported to adopt either monoclinic, tetragonal, or cubic symmetry at room temperature. Using high-resolution synchrotron X-ray diffraction, neutron diffraction and neutron pair distribution function analysis we find that the compound adopts a tetragonal *I*4/*m* double-perovskite structure at room temperature (with a weak, temperature-independent second-order Jahn–Teller distortion in the NbO_6_ octahedra) and undergoes a phase transition to a monoclinic *P*2_1_/*n* symmetry upon cooling to 2.4 K. Only upon heating above room temperature to *T* ≈ 450 K does Ba_2_GdNbO_6_ reversibly transition to a cubic *Fm* 3̅ *m* symmetry. Magnetic susceptibility measurements indicate predominant paramagnetic behavior down to 1.8 K, with minimal ferromagnetic short-range correlations (θ = 0.20(5) K) and a small exchange interaction (*J*_1_ = −0.0032(8) K). At 2 K and 9 T, the compound exhibits a maximum magnetic entropy change of −Δ*S*_m_ = 15.75 J K^–1^ mol^–1^ and an adiabatic temperature change of Δ*T*_ad_ = 21 K, making it a promising candidate for low-temperature magnetocaloric applications. Heat capacity measurements confirm a rigid crystal lattice (*T*_D_ = 267(3) K) and a corresponding small lattice entropy contribution in the low-temperature regime, highlighting the potential of Ba_2_GdNbO_6_ for effective cooling capability in magnetocaloric devices at cryogenic temperatures. This study elucidates the structural and magnetic characteristics of Ba_2_GdNbO_6_ and attests to its promise for low-temperature magnetocaloric refrigeration.

## Graphical abstract

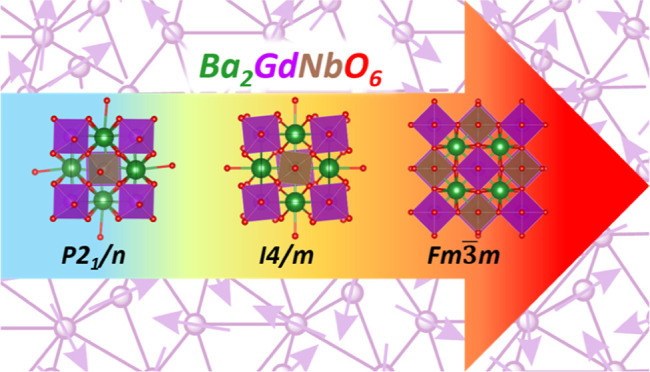

## 1 Introduction

Solid-state cooling technologies are a sustainable alternative to liquid helium for cooling to the cryogenic temperatures required by many technologies, such as magnetic resonance imaging and satellite sensors. Solid-state magnetic refrigeration is based on the magnetocaloric effect (MCE) and is widely recognized as a promising substitute for standard vapor-compression refrigeration due to its high energy efficiency.^1^ The MCE arises from the adiabatic demagnetization of the sample, which causes a temperature decrease as the magnetic spins randomize when the external field is removed, resulting in a magnetic entropy change. Magnetic cooling is limited by the long-range magnetic ordering temperature of the sample; therefore, geometrically frustrated magnets with suppressed ordering temperatures are ideal candidates as working materials in magnetic refrigerators.

Frustrated lanthanide oxides have good magnetocaloric performances due to their large ground state magnetic entropy, suppressed magnetic ordering temperatures, high magnetic moments and minimal nearest neighbor (*nn*) spin interactions. Gadolinium-containing magnets are often top-performing because Gd^3+^ (4f^7^) is isotropic and has no orbital contribution (*L* = 0). The absence of magnetic anisotropy maximizes the entropy change upon the application and removal of an external magnetic field, resulting in Gd^3+^ outperforming other lanthanide ions with single ion anisotropy at high magnetic field.^2–6^

Prior work on gadolinium double-perovskites has shown that they are suitable systems for low-temperature magnetic cooling.^7–9^ In particular, the oxides with general formula *A*_2_ *BB′*O_6_ belong to a vast family of materials that have the potential to exhibit geometric magnetic frustration when *nn* magnetic cations are antiferromagnetically (AFM) correlated.^10–13^ Fully ordered double-perovskites, commonly adopt rock-salt ordering of *B*- and *B′*-site cations, although columnar and layer ordering arrangements are also found.^14–16^ The rock-salt arrangement of the two crystallographically distinct octahedral sites, driven by differences in charge and size of the *B*- and *B′*-site cations, enforces two sets of face-centered-cubic (*fcc*) sublattices, which, in the case of magnetic ions, give rise to magnetic frustration.

Saines *et al.* report a systematic study of the crystal structures across the lanthanide series in Ba_2_ *Ln*SbO_6_ and Ba_2_ *Ln*NbO_6_ (*Ln* = lanthanide).^17^ At room temperature they, and subsequently others,^8^ find that the Ba_2_ *Ln*SbO_6_ series adopts a cubic *Fm* 3̅ *m* structure for *Ln* = Sm–Lu and Y, and rhombohedral *R* 3̅ for *Ln* = La–Nd. For the Ba_2_ *Ln*NbO_6_ series, they report cubic *Fm* 3̅ *m* structures for *Ln* = Ho–Lu and Y, tetragonal *I*4/*m* for *Ln* = Eu–Dy, and monoclinic *I*2/*m* for *Ln* = La, Pr, and Nd, while for *Ln* = Sm they find coexistence of the *I*2/*m* and *I*4/*m* phases at room temperature. They ascribe the increased deviation from the cubic structure in the antimonate and niobate families to the increase in size of the lanthanide ions on the *B*-site and suggest that the empty 4d orbitals of the niobium ions take part in π-bonding with the oxygen anions. This π-bonding can thus explain the differences in symmetry between the antimonate and niobate families, despite the very similar ionic radii of Sb^5+^ and Nb^5+^. Their variable temperature measurements on Ba_2_NdNbO_6_ and Ba_2_SmNbO_6_ reveal phase transitions upon heating above room temperature, incrementally raising the symmetry from monoclinic *I*2/*m* to tetragonal *I*4/*m* and then to cubic *Fm* 3̅ *m*.

Studies have demonstrated that the ionic radius of the *A*-site cation in the gadolinium oxides *A*_2_GdSbO_6_ (*A* = {Ba, Sr, Ca}) affects the crystal structure, which at room temperature is cubic with space group *Fm* 3̅ *m* for Ba^2+^ and monoclinic with space group *P*2_1_/*n* for Sr^2+^.^7,8^ The Ca^2+^ analogue also crystallizes in the monoclinic *P*2_1_/*n* symmetry but features a different cation arrangement, resulting in the site-disordered [CaGd]_*A*_[CaSb]_*B*_O_6_ structure.^7^ There are conflicting results on the room temperature structure of the isovalent Ba_2_GdNbO_6_ analogue with monoclinic *P*2_1_/*n*,^18^ tetragonal *I*4/*m*,^19,20^ and cubic *Fm* 3̅ *m* ^21^ structures proposed. A random arrangement of Gd^3+^ and Nb^5+^ on the *B*-sites with cubic space group *Pm* 3̅ *m* has also been reported.^22,23^ Like for the Sb analogues, the Sr^2+^- and Ca^2+^-containing *A*_2_GdNbO_6_ both adopt the monoclinic *P*2_1_/*n* structure, with the Ca^2+^ phase showing site-disorder.^24,25^

In this study, high-resolution synchrotron powder X-ray diffraction (PXRD) and neutron powder diffraction (NPD) are employed to characterize the structure of Ba_2_GdNbO_6_. We find that Ba_2_GdNbO_6_ is tetragonal (*I*4/*m*) at room temperature, while it undergoes a monoclinic (*P*2_1_/*n*) phase transition upon cooling, and a transition to cubic (*Fm* 3̅ *m*) upon heating. Magnetic measurements show weakly interacting ferromagnetic (FM) correlations between Gd^3+^ spins, no long-range order (LRO) at *T* ≥ 1.8 K and a high MCE of −Δ*S*_m_ = 15.75 J K^–1^ mol^–1^ at 2 K and 9 T.

## 2 Experimental Methods

### 2.1 Solid-State Synthesis

Ba_2_GdNbO_6_ was synthesized via the conventional solid-state method, following a procedure reported in the literature.^7,14^ Stoichiometric amounts of BaCO_3_ (99.997%, Alfa Aesar Puratronic), Gd_2_O_3_ (99.999%, Alfa Aesar REacton) and Nb_2_O_5_ (99.9%, Alfa Aesar) were ground together using a pestle and mortar until a homogeneous mixture was obtained. The powder was then heated in air in a furnace at 1400 °C for 24 h, with a heating rate of 180 °C/h and allowed to cool down naturally. Additional 24 h heating cycles were repeated until no starting materials nor impurity phases were detected by PXRD. A sample of Ba_2_GdNbO_6_ (∼4 g) was successfully synthesized after 48 h and the final product appeared as a white powder. Ba_2_GdSbO_6_ was also synthesized following the method reported in the literature.^7^

### 2.2 X-ray Diffraction

High-resolution synchrotron PXRD data were collected on the I11 beamline at Diamond Light Source,^26,27^ using the MYTHEN-II position-sensitive detector (PSD) at room temperature and at variable temperatures (VT) down to 100 K and up to 950 K. All scans were run with incident photon energy of 15 keV, λ ≈ 0.825 Å (calibrated using a silicon standard), and a 2θ step interval of 0.004°. Borosilicate and quartz capillaries (⌀ = 0.5 mm) were sealed with Araldite Adhesive Bicomponent epoxy, mounted onto brass holders, and continuously rotated during measurement. Rietveld refinements were performed using the DIFFRAC.SUITE TOPAS (V.5 and V.7) program.^28,29^ The Thompson-Cox-Hastings pseudo-Voigt (TCHZ) function was used to model peak shapes,^30^ and a 12-term Chebyshev polynomial was used for the background. Capillary absorption, specimen displacement corrections and strain broadening effects were considered in the refinements.

### 2.3 Neutron Diffraction

NPD data were collected at the Institut Laue-Langevin (ILL) in Grenoble, France, using the D4c diffractometer.^31^ Although gadolinium has a very large thermal neutron cross section (σ_abs_ = 49,700 barn),^32^ short-wavelength neutrons from the hot source guaranteed a high enough transmission to be achieved (*T* = 73%) on the unenriched sample. Diffraction measurements were performed in a cryostat in the temperature range 2.4–300 K with λ = 0.4982 Å (refined using a nickel standard) and an angular definition of ∼0.13° over the 2θ range 1.5° to 137°. The sample powder, with volume of 0.915 cm^3^, was held in a cylindrical Helicoflex-sealed vanadium can (⌀ = 5 mm). The measured diffraction intensity *I*(*Q*) was processed using the Correct software^33^ in order to subtract the background intensity due to the sample environment and to correct the sample’s diffraction intensity for absorption and multiple-scattering. Using the measured packing fraction of the sample powder, and the measured diffraction intensity from a vanadium standard, the sample’s diffraction intensity could then be normalized to an absolute scale of barns/steradian/atom in the form of a differential scattering cross-section dσ/dΩ as a function of the wavevector transfer *Q* (where $Q=\frac{4\pi }{\lambda }\;\sin\;\theta$ ). Rietveld refinements were performed using the DIFFRAC.SUITE TOPAS (V.5 and V.7) program.

### 2.4 Neutron Atomic Pair Distribution Function

The aforementioned differential scattering cross-section dσ/dΩ per atom can be written as^34^

$$\frac{1}{N}\frac{d\sigma }{d\Omega }={\left\langle b\right\rangle }^{2}\left[S\left(Q\right)-1\right]+\langle b^{2}\rangle$$ 1

where *S*(*Q*) is the dimensionless static structure factor approaching 1 for *Q* → ∞, ⟨*b*⟩ is the average coherent scattering length *b* over all atoms in the sample, and the “self-scattering” = ⟨*b* ^2^⟩ is the average of *b* ^2^ over all atoms in the sample. The self-scattering is particularly susceptible to inelastic scattering effects,^35^ and in any case must be subtracted before performing a Fourier transform to obtain the pair distribution function (PDF), PDF­(*r*), also written as *G*(*r*)­

2 $$PDF\left(r\right)=G\left(r\right)=\frac{2}{\pi }\int_{0}^{Q_{\max}}\left[S\left(Q\right)-1\right]Q\;\sin\left(Qr\right)\;dQ$$

where *r* is the interatomic distance. The high *Q*_max_ = 23.5 Å^–1^ of the D4c instrument helps to minimize Fourier truncation artifacts, and it also assures a full-width-at-half-maximum (FWHM) *r*-space resolution of 0.16 Å. Our PDF­(*r*) = *G*(*r*) results were then refined with a small-box modeling approach using the DIFFRAC.SUITE TOPAS (V.7) program.

### 2.5 Magnetic Characterization

Bulk magnetization measurements were performed with a Quantum Design 9 T Physical Property Measurement System (PPMS) using the ACMS-II option. Magnetic susceptibility, χ­(*T*), was measured under zero-field-cooled (ZFC) conditions from 1.8 to 300 K using the direct current (DC) option with applied fields of 0.1, 1, and 2 T. The low-field approximation (χ = ∂*M*/∂*H* ≈ *M*/*H*) was assumed to hold over this field range. Isothermal magnetization, *M*(*H*), measurements were run at 2, 4, 6, 8, 10, 30, and 300 K, from 0 to 9 T, in steps of 0.1 T.

The magnetic entropy change, Δ*S*_m_, an effective way of evaluating the magnetocaloric performance of a material, was extracted from the *M*(*H*) data by applying one of Maxwell’s relations

3 $${\left(\frac{\partial S_{m}}{\partial H}\right)}_{T}={\left(\frac{\partial M}{\partial T}\right)}_{H}$$

Integration of eq 3 allows calculation of Δ*S*_m_ at temperature *T*_0_ for an increase in field from zero to *H*_max_ from bulk DC magnetic measurements. Prior to integration, the *M*(*H*) data were linearly resampled at uniform μ_0_ *H* intervals of ∼0.4 T to suppress high-frequency noise and prevent spurious oscillations in the Δ*S*_m_ integral

4 $$\Delta S_{m}=\int_{0}^{H_{\max}}{\left(\frac{\partial M}{\partial T}\right)}_{H}dH$$

### 2.6 Heat Capacity

Heat capacity (HC) was measured with a Quantum Design 9 T PPMS in the temperature range 1.8–50 K in zero-field. The sample (0.2234 g) was ground with silver powder (spherical, −635 mesh, 99.9%, Alfa Aesar) in a 50:50 mass ratio to enhance thermal conductivity,^36^ before being pressed into a pellet (⌀ = 8 mm). A small fragment of the pellet (13.08 mg) was mounted onto the sample platform using a thin layer of Apiezon N grease to ensure good thermal contact. An addenda measurement of the sample platform and grease was run before the measurement. The silver contribution was subtracted from the total measured HC, *C*_tot_, using literature values,^37^ to obtain the specific heat contribution of the sample at constant pressure, *C*_p_. The magnetic HC, *C*_m_, was determined by subtracting the lattice phonon contribution, *C*_lat_, from *C*_p_. The phonon contribution, *C*_lat_, was estimated using least-squares fits of the zero-field *C*_p_ at high temperatures (39–50 K) to the Debye model^38^

5 $$C_{lat}=\frac{9nRT^{3}}{T_{D}^{3}}\int_{0}^{T_{D}/T}\frac{x^{4}e^{x}}{{\left(e^{x}-1\right)}^{2}}\;dx$$

where *n* is the number of atoms per formula unit, *R* is the molar gas constant, and *T*_D_ is Debye temperature. The total magnetic entropy, relative to the lowest temperature *T*_*i*_ measured, was computed using

6 $$S_{m}\left(T,\Delta H\right)=\int_{T_{i}}^{T}\frac{C_{m}\left(T^{\prime },H\right)}{T^{\prime }}\;dT^{\prime }$$

## 3 Results and Discussion

### 3.1 Structure of Ba_2_GdNbO_6_

#### 3.1.1 Room Temperature Structure

PXRD and NPD confirmed formation of phase-pure Ba_2_GdNbO_6_. A combined Rietveld refinement was conducted on room temperature synchrotron PXRD and NPD data (Figure 1). While the resolution of the NPD is much lower than the PXRD, NPD is more sensitive to oxygen position, hence complementary structural information was obtained from the combined refinement. All four of the previously reported structural models were refined against our data, see Table S1 and Figure S1 in the Supporting Information, and we find that the tetragonal *I*4/*m* structure provides the best fit to the data. Refinements including Gd/Nb antisite disorder give a small value for the antisite disorder, 0.043(4), and a negligible change in global fit (*R*_wp_ improves from 4.6616% to 4.6606%, and χ^2^ from 5.6218 to 5.6205). Hence, we consider the *B*- and *B′*-sites predominantly rock-salt ordered, and we use the fully ordered model for all subsequent analyses, we note that this small degree of antisite disorder does not affect our conclusions. We therefore conclude that Ba_2_GdNbO_6_ crystallizes in the predominantly rock-salt ordered double-perovskite structure with tetragonal symmetry and space group *I*4/*m* at room temperature (Table 1). This structure allows for octahedral rotations described by Glazer notation *a* ^0^ *a* ^0^ *c* ^–^, i.e. no octahedral tilts about the [100] and [010] pseudocubic axes and out-of-phase tilting along the [001] direction.^39,40^ The almost-full rock-salt arrangement of the two crystallographically distinct octahedral sites enforces the gadolinium *fcc* sublattice. However, due to the tetragonal symmetry, the edge-sharing tetrahedra have nonuniform side lengths, Figure 2.

**Figure 1:**
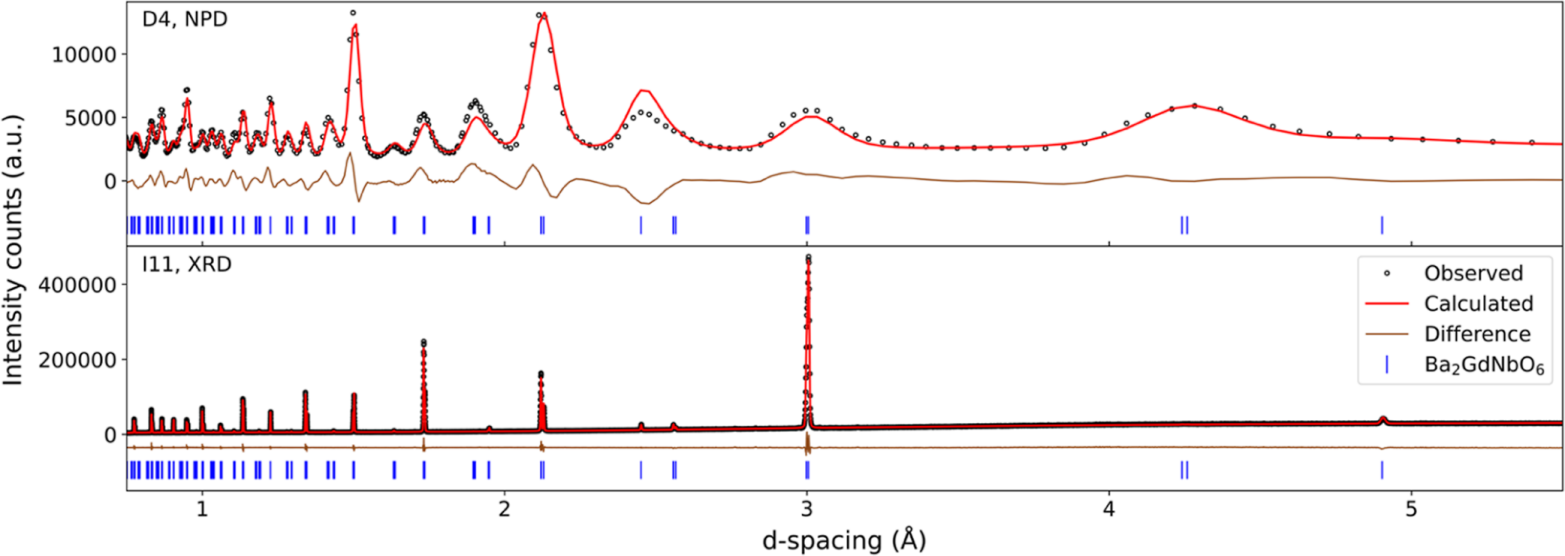
Combined Rietveld refinement of room temperature synchrotron PXRD and NPD. Open black circles correspond to experimental data points, the red line to the calculated Rietveld fit, the brown line to the difference between the two, and the blue tick marks to Bragg reflection positions.

**Table 1:** Structural Parameters and Wyckoff Positions of Ba_2_GdNbO_6_ Obtained from Combined Rietveld Refinement of Room Temperature PXRD and NPD in the Space Group *I*4/*m*

*R*_wp_ = 4.66%; χ^2^ = 5.62					
*a* = *b* = 5.996284(8) Å, *c* = 8.515386(18) Å, volume = 306.1743(15) Å^3^					
atom	Wyckoff position	*x*	*y*	*z*	*B*_iso_ (Å^2^)
Ba	4*d*	0	0.5	0.25	1.67(4)
Gd	2*a*	0	0	0	0.13(3)
Nb	2*b*	0	0	0.5	0.37(5)
O(1)	4*e*	0	0	0.2689(4)	0.43(4)
O(2)	8*h*	0.2392(7)	0.2926(7)	0	4.08(10)

**Figure 2:**
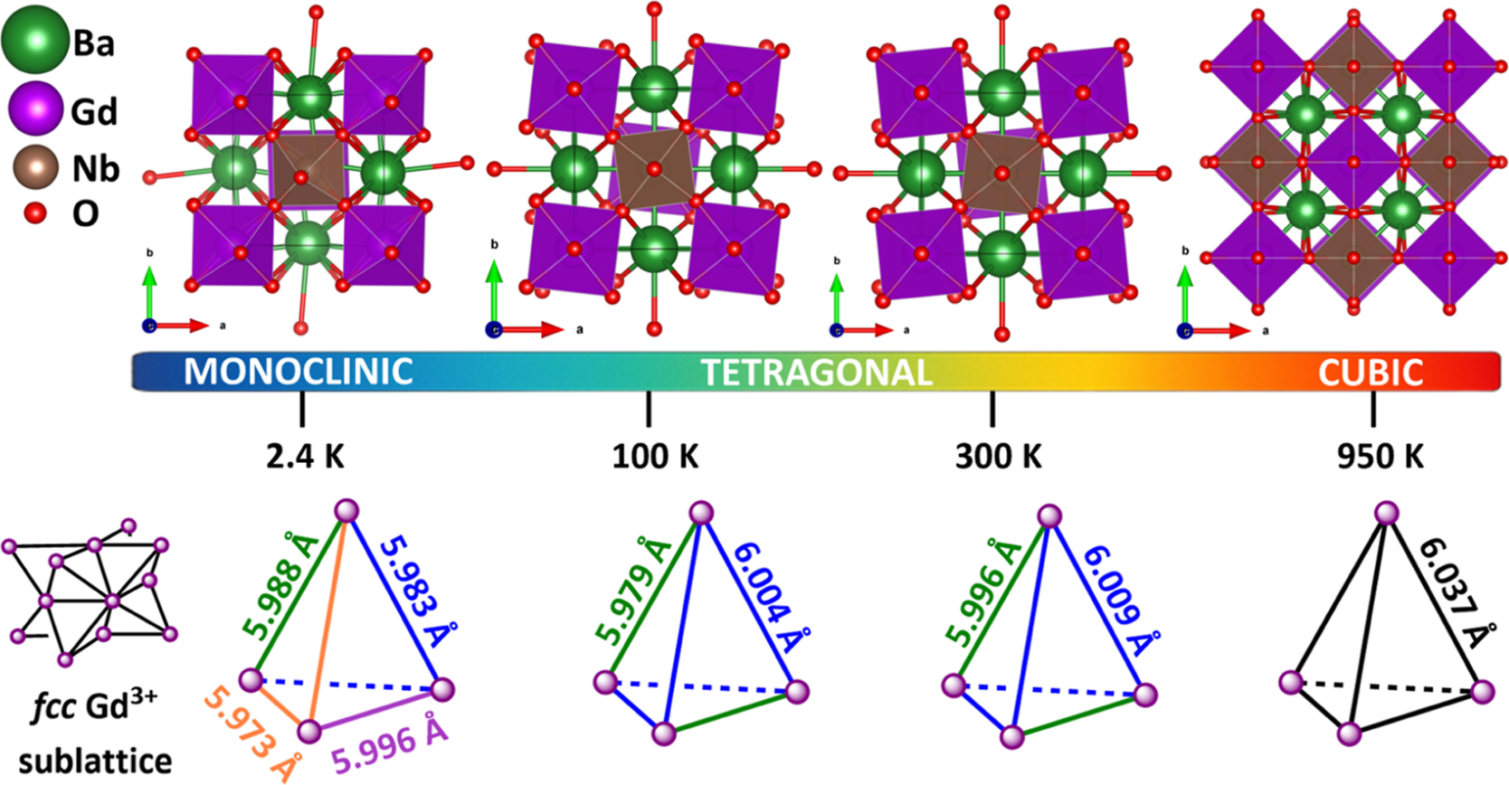
Temperature evolution of the crystal structure of Ba_2_GdNbO_6_ viewed along the *c* axis (top) and its frustrated *fcc* Gd^3+^ sublattice with bond length variations (bottom).

In addition to PXRD and NPD, neutron PDF analysis was performed to compare the average structure to the local structure. Structural information obtained from the combined PXRD and NPD refinement was used as initial parameters for the small-box modeling of neutron PDF data, and then lattice parameters, atomic positions, site occupancy and thermal factors were refined. Structural parameters extracted from PDF data were in good agreement with those derived from the Rietveld analysis. The PDF analysis confirms that the average structure of the sample is tetragonal with space group *I*4/*m* at 300 K (Figure 3a); however, there are peaks in the low-*r* region that are poorly fit by the *I*4/*m* space group suggesting that local environment breaks the average tetragonal symmetry. Further refinements were conducted to model local deviations from the average structure. We found that the tetragonal model does not fully describe the local structure (Figure 3b). Lowering the symmetry to monoclinic, improves the fit at low-*r*. This suggests that the local structure, *r* < 5.9 Å, is better described as monoclinic with space group *P*2_1_/*n* (Figure 3c) where tilting of octahedra in all three pseudocubic axes is allowed rather than only rotation about the [001] pseudocubic axes in the average tetragonal structure. We estimate a correlation length of ∼6 Å for the monoclinic distortions at 300 K, i.e. correlated over 2–3 octahedra or *nn* Gd^3+^ interactions. In the *r*-range 1–17 Å, the monoclinic structure is a poor fit to the data, with the *I*4/*m* structure still providing a better fit. This is similar to the reported order–disorder model observed in BaTiO_3_ and other perovskites.^41–43^ Figure S2 shows the evolution of the monoclinic distortions over increasing length scales when performing a box-car analysis over fixed *r*-ranges of 6 Å. In this case, the monoclinic symmetry fits better than the tetragonal symmetry at all length scales due to the limited resolution of the D4c diffractometer for crystalline materials, group–subgroup relation between *I*4/*m* and *P*2_1_/*n* and more degrees of freedom. However, the monoclinic distortion metric (*R*_ab_ = *a*_mon_/*b*_mon_) shows that the lattice parameters of the monoclinic cell essentially converge to tetragonal symmetry at longer length scales, indicating that at high-*r* the structure is most appropriately described as tetragonal (see details in Section 1.2 in the Supporting Information).

**Figure 3:**
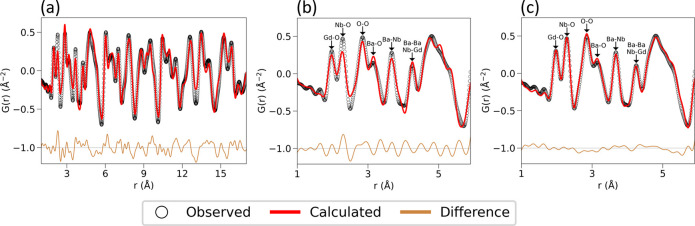
Small-box modeling of neutron PDF data of Ba_2_GdNbO_6_ at 300 K of (a) average structure in the tetragonal *I*4/*m* space group up to 17 Å; (b) local structure in the tetragonal *I*4/*m* space group and (c) in the monoclinic *P*2_1_/*n* space group up to 5.9 Å. *r*_min_ was 1 Å for all refinements. Experimental data points (open black circles) and refined models (red lines) are shown, along with the difference pattern (brown line).

#### 3.1.2 Changes to the Structure with Heating and Cooling

VT-PXRD (*T* = 100–950 K) and NPD (*T* = 2.4–300 K, see Figure S3 for NPD patterns) data were analyzed to investigate structural phase transitions in Ba_2_GdNbO_6_. Changes in temperature cause significant changes in the VT-PXRD diffraction patterns with both peak broadening and splitting observed on cooling (Figure 4a). At the highest measured temperature, *T* = 950 K, a cubic structure is observed, with the symmetry reducing to tetragonal on cooling due to the formation of octahedral tilt ordering between 460.75 and 445.95 K. The continuous changes in the lattice parameters and no coexistence of the cubic and tetragonal phases are consistent with a second-order phase transition. Inspection of the VT-PXRD data shows clear splitting of the (*hkl*)_cubic_ peaks below 445 K, and the degree of tetragonal distortion, $Dist_{t}=\frac{\left(\frac{c_{t}}{\sqrt{2}}-a_{t}\right)}{\frac{1}{2}\left(\frac{c_{t}}{\sqrt{2}}+a_{t}\right)}\%$ where the subscript *t* stands for tetragonal, increases continuously upon cooling, with no evidence of further symmetry lowering to the monoclinic structure at the lowest temperature measured, *T* = 100 K (Figure S4). A heatmap showing the evolution of the (022)_cubic_ reflection into the (112)_tetragonal_ and (200)_tetragonal_ reflections in the VT-PXRD data is shown in Figure 4b. We observe that both the phase transition and increased tetragonal distortion are fully reversible (Figure S5). The refined lattice parameters, scaled to the high-symmetry cubic structure, the volume/*f.u.*, and the degree of tetragonal distortion as a function of temperature are presented in Figure 4c and tabulated for selected temperatures in Tables S2 and S3. At all temperatures, Ba_2_GdNbO_6_ undergoes positive thermal expansion, with the negative thermal expansion in *c*_t_ offset by thermal expansion in *a*_t_ = *b*_t_. Anisotropic thermal expansion is often observed in layered oxides,^44–46^ and here can be ascribed to the octahedral tilting in *c*_t_ allowed in the tetragonal structure (we note that its lattice parameters correspond to the principal axes, Table S4 and Figure S6).

**Figure 4:**
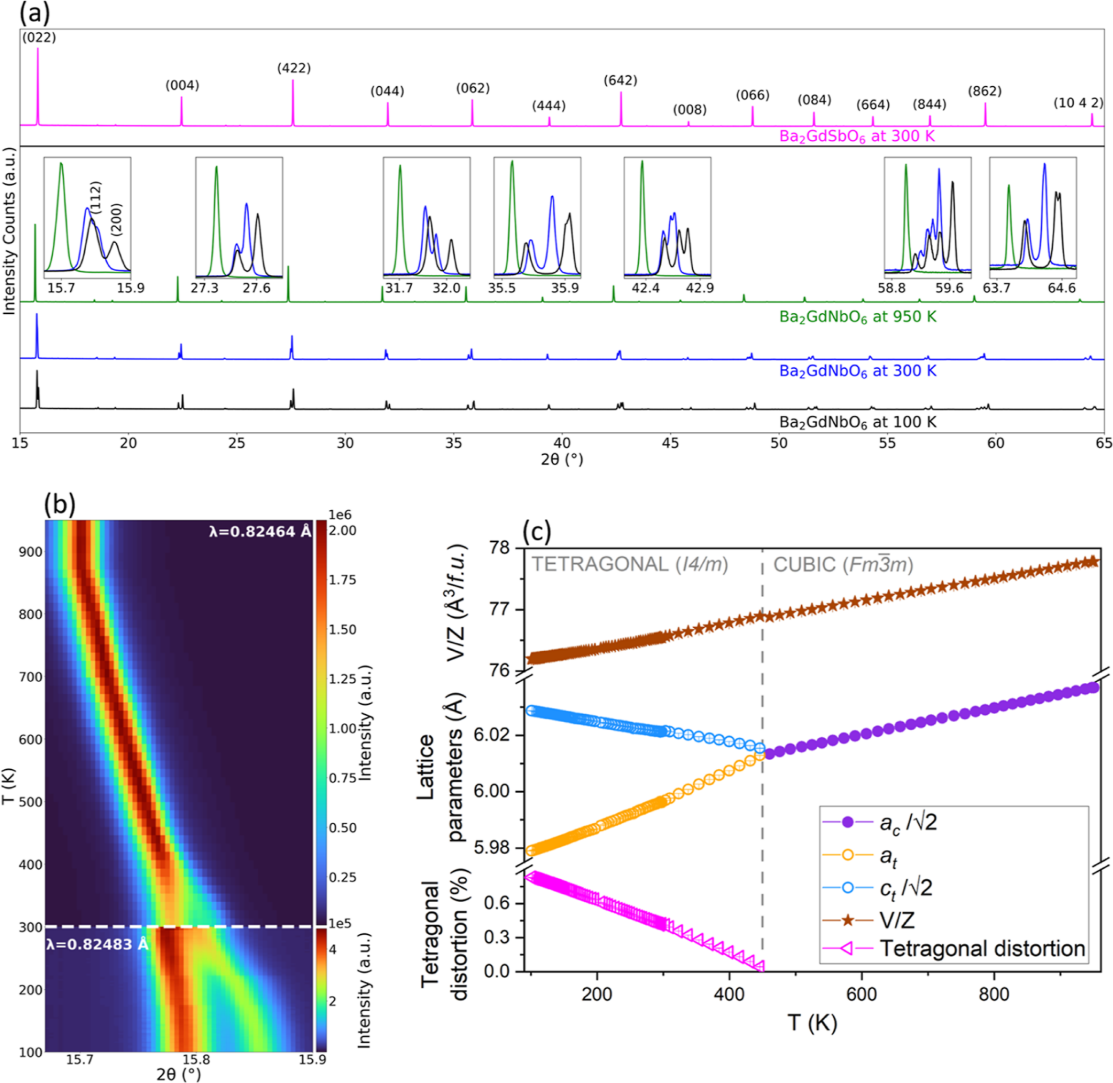
(a) Synchrotron VT-PXRD data showing the structural transformation of Ba_2_GdNbO_6_, undergoing a cubic (*Fm3m*) to tetragonal (*I*4/*m*) phase transition upon cooling. Zoomed in regions are shown to highlight peak splitting. The PXRD pattern of the Ba_2_GdSbO_6_ analogue at room temperature is included for reference; (b) Heatmap of intensity in VT-PXRD of the most intense (022)_cubic_ peak in Ba_2_GdNbO_6_ as a function of 2θ and temperature in the range 950–100 K. Data were collected with λ = 0.82464 Å (top) and λ = 0.82483 Å (bottom); (c) variation of lattice parameters, volume/*f.u.*, and degree of tetragonal distortion over the temperature range 950–100 K, as determined from VT-PXRD. The error bars are smaller than the data points.

The changes in the crystal structure of Ba_2_GdNbO_6_ on cooling below 100 K were examined by VT-NPD measurements. Due to the continuous nature of the phase transition between the tetragonal and monoclinic structures it is not possible to identify the transition temperature from only the D4c data and higher resolution data is required. Inspection of both the NPD and neutron PDF data set at 2.4 K indicates that the sample is monoclinic over all length scales, with no further symmetry lowering in the local environment, and is best fit by the monoclinic *P*2_1_/*n* symmetry (Figures 5 and S7 and Table S5), confirming that a tetragonal to monoclinic transition occurs upon cooling below 100 K. This transition leads to antiphase tilting along the [100] and [010] directions of the pseudocubic cell and in-phase tilting about the [001] axis or *a* ^–^ *a* ^–^ *c* ^+^ using Glazer notation. The same changes in octahedral tilting are observed in the double-perovskite Sr_2_CoWO_6_, which undergoes a tetragonal to monoclinic phase transition at 260 K.^47^ The octahedral rotations affect the *fcc* magnetic sublattice, splitting the Gd^3+^–Gd^3+^ bond lengths into four distinct values (Figure 2) and thereby changing the superexchange interactions.

**Figure 5:**
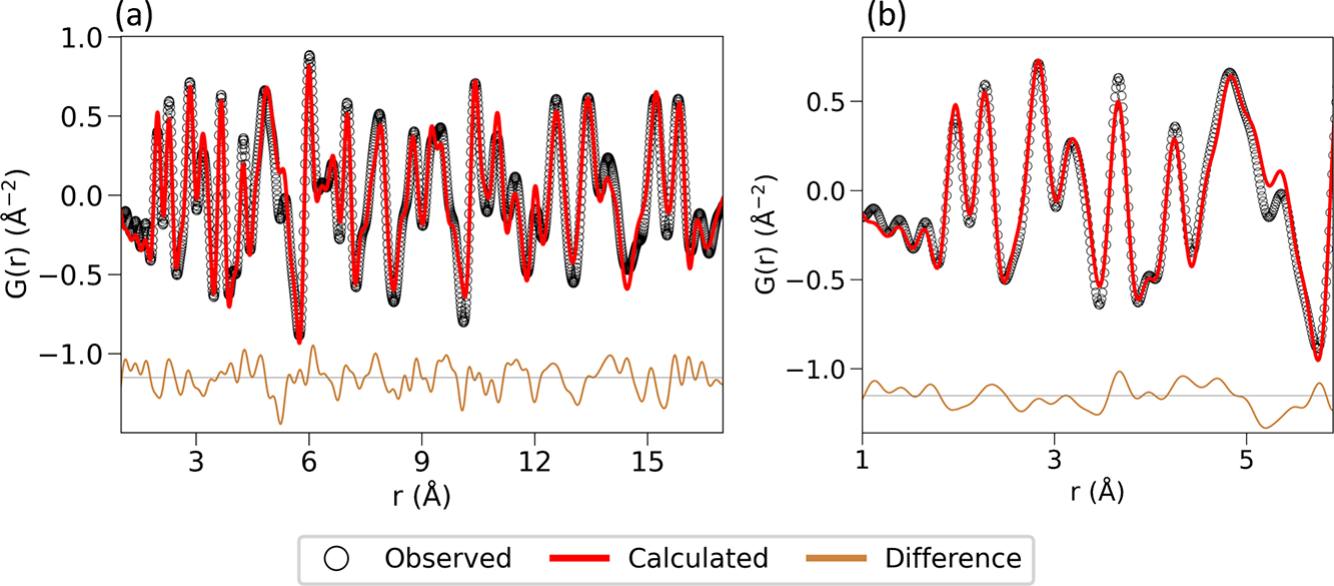
Small-box modeling of neutron PDF data of Ba_2_GdNbO_6_ at 2.4 K of (a) average structure up to 17 Å and (b) local structure up to 5.9 Å, both carried out in the monoclinic *P*2_1_/*n* space group. *r*_min_ was 1 Å for both refinements. Experimental data points (open black circles), refined models (red lines), and difference patterns (brown lines) are shown.

#### 3.1.3 Second-Order Jahn–Teller Effect

Due to the d^0^ nature of Nb^5+^ ions, the presence of second-order Jahn–Teller (SOJT) distortion cannot be excluded *a priori* in Ba_2_GdNbO_6_.^48–50^ We use neutron PDF to assess the presence of cation off-centering with respect to the oxygen anions in the NbO_6_ octahedra associated with a SOJT distortion. A distortion is not prominent from a visual inspection of the PDF patterns with no peak splitting of the Nb–O bond at ∼2.3 Å (Figures 3c and 5b). To explore a possible SOJT distortion more quantitatively, refinements of the PDF local structure at all measured temperatures (2.4, 3.1, 5, 10, 30, 42, 50, 60, and 300 K) were performed using the triclinic *P*1 space group to allow for the off-centering of the Nb^5+^ ion, while constraining other sites and lattice parameters to follow *P*2_1_/*n* symmetry. The off-centering distance between the center of the O_6_ polyhedron and the Nb^5+^ cation position, *d*_oct_, was calculated using the following formula^51^

7 $$d_{oct}=∥r_{Nb}-\sum_{i=1}^{6}\frac{r_{O,i}}{6}∥$$

where *r*_Nb_ is the position of the Nb^5+^ cation and *r*_O,*i*_ are the positions of the oxygen anions. The magnitude of the SOJT distortion, Δ_d_, defined by Halasyamani,^52^ was also calculated (see details in the Supporting Information, Equation S1 and Figure S8). When *d*_oct_, Δ_d_ ≠ 0, the octahedron deviates from its ideal structure, indicating a degree of off-centering and a SOJT distortion. The calculation was performed using the DIFFRAC.SUITE TOPAS (V.7) program and the Python package *VanVleckCalculator*.^53^ At all temperatures both Nb positions in the expanded triclinic cell were found to have octahedral distortions, with only minor changes as a function of temperature, Figure S9 and Table S6. The off-centering distance at 300 K (2.4 K) was calculated to be 0.090(8) Å (0.082(10) Å) for the Nb1 site and 0.089(7) Å (0.082(8) Å) for the Nb2 site. Consideration of Δ_d_ enables the degree of the SOJT to be evaluated. The values obtained (Δ_d_Nb1__ = 0.23(5) and Δ_d_Nb2__ = 0.15(4) at 300 K; and Δ_d_Nb1__ = 0.20(7) and Δ_d_Nb2__ = 0.13(6) at 2.4 K) fall in the weak distortion range (0.05 < Δ_d_ < 0.40), consistent with the presence of a weak SOJT effect at all measured temperatures. Further studies, beyond the scope of this work, are required to fully understand the origin of the SOJT distortion in Ba_2_GdNbO_6_ and to determine whether it is static or dynamic in nature.

### 3.2 Magnetic and Magnetocaloric Analysis

Magnetic susceptibility measurements indicate that Ba_2_GdNbO_6_ exhibits paramagnetic behavior down to 1.8 K with no evidence of magnetic ordering transitions (Figure 6a), similar to the antimonate analogue.^7,14^ Fitting to the Curie–Weiss Law, *T* = 8–50 K, at 0.1 T indicates weak FM exchange with θ = 0.20(5) K and μ_eff_ = 7.996(17) μ_B_, as expected for *J* = *S* = 7/2 4f^*7*^ Gd^3+^ (μ = 7.94 μ_B_). The geometric frustration was estimated using the frustration index *f* = |θ|/*T*_0_, where *T*_0_ was taken as the lowest measured temperature due to lack of magnetic ordering,^54^ giving *f* > 0.1. Further measurements in the millikelvin regime are required to determine *T*_0_ and precisely quantify the degree of frustration of the magnetic lattice. Rearranging the Curie–Weiss law $\chi =\frac{C}{T-\theta }$ , to its dimensionless form $\frac{C}{\left|\theta \right|\chi }=\frac{T}{\left|\theta \right|}\pm 1$ , allows us to assess the presence of short-range correlations in more detail. Here, ideal Curie–Weiss behavior corresponds to a straight line with a *y*-intercept of −1 for θ > 0.^55,56^ Negative deviations from the ideal Curie–Weiss behavior are evidence of FM fluctuations or short-range order, while positive deviations indicate AFM correlations. Ba_2_GdNbO_6_ shows minimal deviations down to 1.8 K (Figure 6b), consistent with the absence of magnetic diffuse scattering in neutron diffraction patterns even at 2.4 K (Figure S7). This indicates that, while the small positive Curie–Weiss temperature reflects weak net FM exchange interactions, there are no significant deviations from Curie–Weiss behavior above 1.8 K.

**Figure 6:**
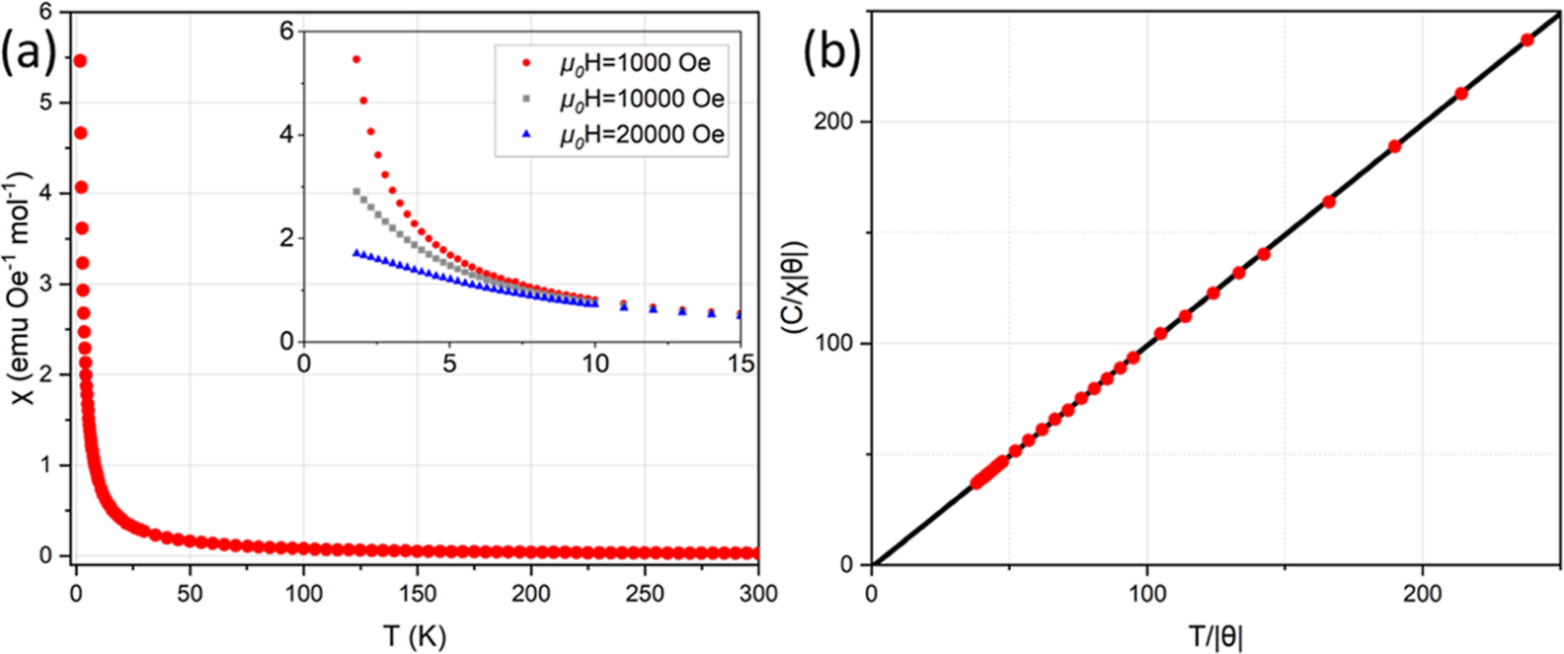
(a) ZFC magnetic susceptibility, χ­(*T*), of Ba_2_GdNbO_6_ in a field of 1000, 10,000, and 20,000 Oe. The inset shows the zoomed in region below 15 K. (b) Dimensionless inverse magnetic susceptibility, *C*/χ|θ|, versus *T*/|θ|. The straight line corresponds to ideal Curie–Weiss behavior for θ > 0.

Gd^3+^ has half-filled 4f orbitals with no contribution from angular momentum (*L* = 0) to the expected moment. We can therefore assume Heisenberg-like behavior and use a mean-field analysis to obtain more information on the magnetic interactions.^7^ A mean-field estimate for the *nn* superexchange *J*_1_ = −(3θ/*zS*(*S* + 1)), where *z* is the number of *nn* ions for a single magnetic ion (*z* = 12 in *fcc* lattices) and *S* is the total spin quantum number, indicates that Ba_2_GdNbO_6_ exhibits exceptionally weak coupling between the spins (*J*_1_ = −3.2(8) mK). This is significantly smaller than the reported *J*_1_ for the antimonate analogue (12.4(2) mK),^7^ and for the top-performing gadolinium gallium garnet (GGG), Gd_3_Ga_5_O_12_, whose *J*_1_ values range from 107 mK to 510 mK.^6,7,57^ Similarly, a mean-field analysis can be used to obtain the dipolar coupling between spins, *D* = *D*_*nn*_/(*S*(*S* + 1)) = 11.68(2) mK (where $D_{nn}=\frac{\mu_{0}\mu_{eff}^{2}}{4\pi R_{nn}^{3}k_{B}}$ , μ_0_ is vacuum permeability, *R*_*nn*_ is the distance between *nn* magnetic ions, and *k*_B_ is Boltzmann’s constant). Although still small, the dipolar coupling is significantly higher than the calculated *J*_1_, therefore both need to be considered when describing the magnetic behavior of the compound. The results for Ba_2_GdNbO_6_ are compared to the other Gd^3+^ systems in Table 2. The different sign of *J*_1_ between these systems highlights the difference in the magnetic interactions, with *J*_1_ < 0 indicating ferromagnetic coupling in Ba_2_GdNbO_6_. Moreover, unlike Ba_2_GdSbO_6_ and GGG, the dipolar coupling in Ba_2_GdNbO_6_ exceeds the *nn* superexchange (*D* > *J*_1_), playing the dominant role in the low-temperature magnetic behavior of the compound. These differences suggest a different interplay of interactions in the niobate analogue, with implications for its low-temperature magnetic and magnetocaloric properties.

**Table 2:** Curie–Weiss Temperature θ, Experimental Effective Magnetic Moment μ_eff_, Estimate for the Mean-Field *nn* Exchange *J*_1_, Dipolar Interaction *D*, and Frustration Index *f* Obtained From the Curie–Weiss Fit for Ba_2_GdNbO_6_, Compared to the Values Reported for the Antimonate Analogue and the Top-Performing GGG

material	θ (K)	μ_eff_ (μ_B_/*f.u.*)	*J*_1_ (K)	*D* (K)	*D*/*J*_1_	*f*>
**Ba**_**2**_ **GdNbO**_**6**_	0.20(5)	7.996(17)	–0.0032(8)	0.01168(2)	–3.65	0.1
Ba_2_GdSbO_6_ ^7,58^	–0.78(1)	8.18(1)	0.0124(2)	0.0116	0.94	0.4
Gd_3_Ga_5_O_12_ ^6,54,57,59^ ^a^	–2.3	7.814(3)	0.107	0.0457	0.43	90

a Literature reports of magnetism in Gd_3_Ga_5_O_12_ are highly sample dependent.

Isothermal magnetization measurements for Ba_2_GdNbO_6_ are shown in Figure 7. We model isothermal magnetization using the Brillouin function, *B*_J_(*y*), which describes the behavior of free Heisenberg spins^60^

8 $$B_{J}\left(y\right)=\frac{2J+1}{2J}\;\coth\left(\frac{2J+1}{2J}y\right)-\frac{1}{2J}\;\coth\left(\frac{y}{2J}\right)$$

**Figure 7:**
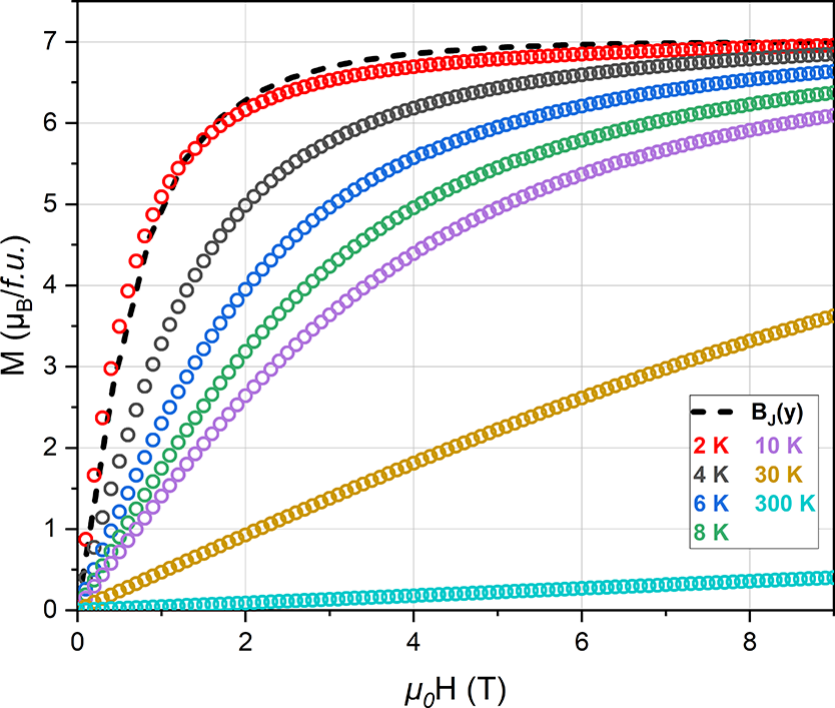
Isothermal magnetization, *M*(*H*), of Ba_2_GdNbO_6_ at selected temperatures. The Brillouin function describing the paramagnetic free Heisenberg spins behavior is shown as a black dashed line.

The *M*(*H*) at 2 K is well described by the Brillouin function, highlighting that FM correlations are minimal. Ba_2_GdNbO_6_ reaches a maximum saturation of *M* ≈ 6.96 μ_B_/*f.u.*, in agreement with the expected value of 7 μ_B_/*f.u.* for fully aligned Gd^3+^ spins. A five-loop isothermal magnetization measurement at 2 K (field sweep: 0 T → 9 T → −9 T → 9 T) reveals no magnetic hysteresis, indicating full reversibility of magnetization/demagnetization cycles (Figure S10).

The magnetic entropy change, −Δ*S*_m_, was calculated from the *M*(*H*) measurements. Here we evaluate it per mole of Gd^3+^ because this allows us to better investigate the role of superexchange in the magnetocaloric effect and directly compare the intrinsic magnetic properties across different materials. −Δ*S*_m_ of Ba_2_GdNbO_6_ in gravimetric (J K^–1^ kg^–1^) and volumetric (mJ K^–1^ cm^–3^) units is given in Table S7. Ba_2_GdNbO_6_ exhibits a maximum magnetic entropy change of −Δ*S*_m_ = 15.75 J K^–1^ mol^–1^ at 2 K and 9 T (Figure 8a), similar to the Ba_2_GdSbO_6_ analogue with 15.84 J K^–1^ mol^–1^,^7^ and outperforming GGG with 14.12 J K^–1^ mol^–1^.^6^ Having three Gd atoms per formula unit, GGG is however better performing when estimating the magnetic entropy change in gravimetric or volumetric units. The maximum magnetic entropy change achieved by Ba_2_GdNbO_6_ at 2 K and 9 T corresponds to ∼91% of the −Δ*S*_max_ = *R* ln­(2*J* + 1) = 17.29 J K^–1^ mol^–1^ predicted for free Heisenberg spins (Figure 8b), consistent with the low Curie–Weiss temperature.

**Figure 8:**
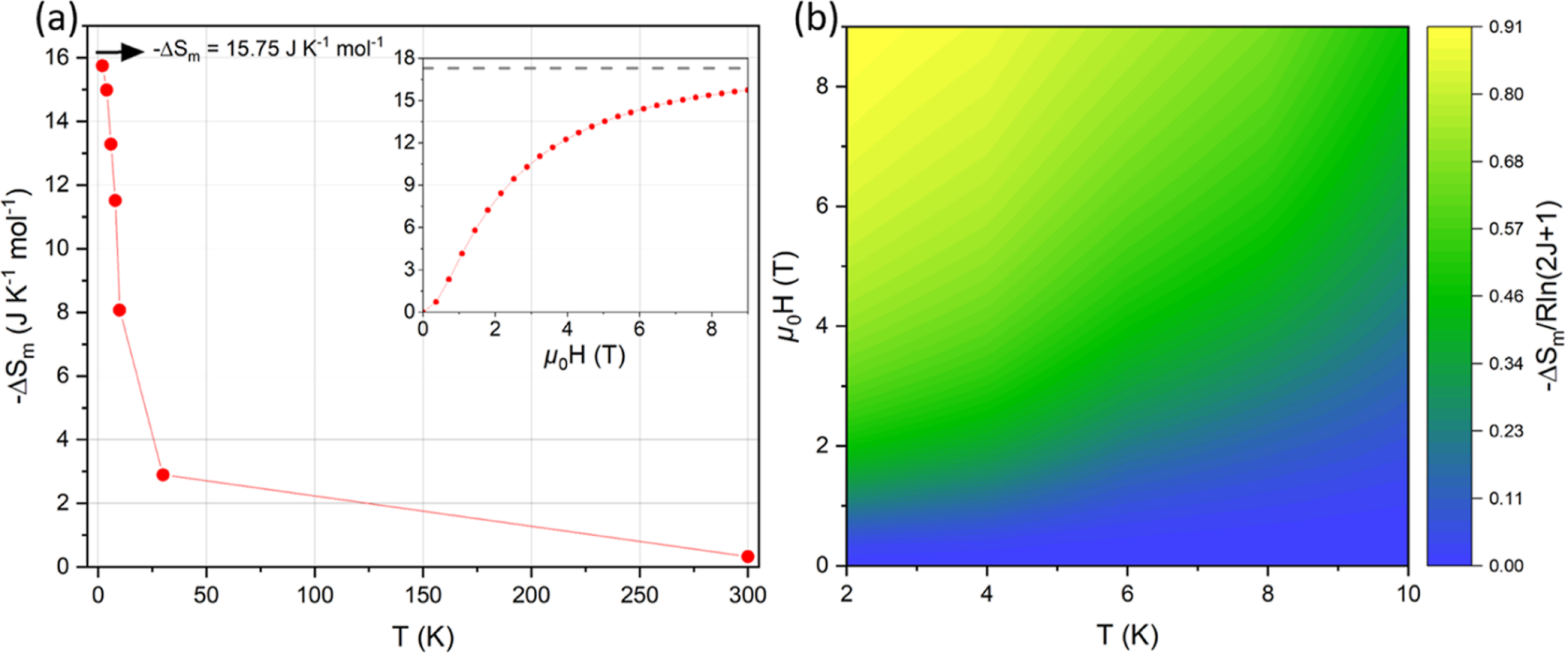
(a) Magnetic entropy change, −Δ*S*_m_, of Ba_2_GdNbO_6_ versus temperature in a field of 9 T. The inset shows −Δ*S*_m_ versus field at *T* = 2 K. The gray dashed line delimits the maximum change −Δ*S*_max_ = 17.29 J K^–1^ mol_Gd_^–1^. Measured values are shown as data points, solid lines are shown to aid the eye. (b) Heatmap of −Δ*S*_m_ as a function of temperature and field, normalized by −Δ*S*_max_ = *R* ln (2*J* + 1). The color bar is scaled to a maximum value of 0.91.

### 3.3 Heat Capacity

Figure 9a shows the zero-field magnetic HC of Ba_2_GdNbO_6_ normalized by temperature, *C*_m_/*T*, after subtraction of the lattice contribution using the Debye model (eq 5, Debye temperature *T*_D_ = 267(3) K, error considers a mass uncertainty of ±0.1 mg), and the magnetic entropy, *S*_m_. The high Debye temperature indicates a rigid crystal lattice and a corresponding small lattice entropy in the low-temperature regime, favoring the MCE, hence making the compound an ideal candidate for magnetocaloric applications.^4,7,61^ The magnetic entropy *S*_m_, computed from the HC measurement by integrating *C*_m_/*T*, at 10 K accounts for ∼83% of the total entropy, while the lattice component contributes to the remaining ∼17% (Figure 9b). At 10 K, *S*_m_ corresponds to only ∼4% of that available for *S* = 7/2 Heisenberg spins. The low value is in line with that reported for the Ba_2_GdSbO_6_ analogue in zero-field^7^ and with the absence of significant magnetic correlations at *T* > 1.8 K. At 50 K, the total entropy is ∼23.5 J K^–1^ mol^–1^, of which ∼7.9 J K^–1^ mol^–1^ (∼34%) is magnetic and ∼15.6 J K^–1^ mol^–1^ (∼66%) comes from the lattice. The narrow asymmetric maximum in the magnetic HC at 3.74 K can be attributed to magnetic ordering of a small percentage of the precursor Gd_2_O_3_ (<1 wt%, below the detection limit of synchrotron PXRD), as its magnetic ordering temperature is 3.8 K.^62^ On further cooling, there is a steep rise in the HC suggesting magnetic ordering at *T* < 1.8 K. There is no evidence of any other anomaly or phonon bottleneck at low-temperature.

**Figure 9:**
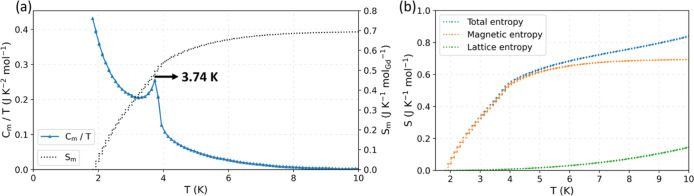
(a) Zero-field magnetic HC of Ba_2_GdNbO_6_ normalized by temperature, *C*_m_/*T* (blue line), and corresponding magnetic entropy, *S*_m_ (dotted black line), from 1.8 to 10 K. (b) Entropy from 1.8 to 10 K. Total entropy is ∼0.83 J K^–1^ mol^–1^ (blue line), of which ∼0.69 J K^–1^ mol^–1^ is magnetic (orange line) and ∼0.14 J K^–1^ mol^–1^ comes from the lattice (green line).

### 3.4 Discussion

Using high-resolution synchrotron PXRD combined with NPD and neutron PDF analysis we are able to study the structure of Ba_2_GdNbO_6_ between 2.4 and 950 K. At room temperature, we find that the average structure is tetragonal (*I*4/*m*) with short-range, *r* < 6 Å, monoclinic distortions. On cooling, the structure distorts and at 2.4 K both the average and local structures are monoclinic (*P*2_1_/*n*). On heating above ∼450 K a cubic structure (*Fm* 3̅ *m*) is observed. Structural phase transitions in perovskites are not uncommon with Saines *et al.* reporting similar behavior for Ba_2_NdNbO_6_ and Ba_2_SmNbO_6_.^17^ However, this contrasts dramatically with the Ba_2_GdSbO_6_ analogue, which is found to be cubic at room temperature.^7^ The Goldschmidt tolerance factor (GTF), *t*, is one way to explore expected distortions in perovskite and double-perovskite systems.^63^ For *A*_2_ *BB′*O_6_ double-perovskites, the GTF formula can be modified to account for the different *B*- and *B′*-sites to^64^

$$t=\frac{r_{A}+r_{O}}{\sqrt{2}\left(r_{B,avg}+r_{O}\right)}$$ 9

where *r*_*A*_ and *r*_O_ are the ionic radii of the *A*-site cation and O, respectively; *r*_*B*,avg_ is an average of the *B*- and *B′*-site cations radii; with *t* ≈ 1 indicating a stable cubic double-perovskite structure. The similarity between the ionic radius of Nb^5+^ and Sb^5+^ means that the GTFs for Ba_2_GdNbO_6_ and Ba_2_GdSbO_6_ are comparable, 0.97 and 0.98, respectively. Likewise, the octahedral factor, μ = *r*_*B*,avg_/*r*_O_, of μ = 0.56 and μ = 0.54, for Ba_2_GdNbO_6_ and Ba_2_GdSbO_6_, respectively, predicts stable cubic perovskite structures.^65^ It is therefore likely that there is something beyond size effects driving the Nb and Sb analogues to adopt different structures.

Differences between the structure of Nb and Sb systems are observed in many oxides, and this is frequently discussed in relation to the 4d^0^ versus 4d^10^ electronic configuration for Nb^5+^ and Sb^5+^, respectively, with the former configuration allowing π-bonding with the 2p orbitals of the oxygen anions.^17,66^ Nb^5+^ oxides are also observed to form hexagonal perovskites with both face and corner sharing octahedra more frequently than the Sb^5+^ analogues.^67^ In the case of Ba_2_GdNbO_6_ and Ba_2_GdSbO_6_, *t* < 1 and hence the hexagonal perovskite structures are not stable. Instead, in Ba_2_GdNbO_6_, we find a weak, temperature-independent SOJT distortion and symmetry lowering from the cubic to the tetragonal and then monoclinic structure upon cooling. However, it is important to note that this same pattern of phase transitions is observed in other double-perovskites without SOJT distortions.^17,68^ Structural differences between similar-size d^10^ and d^0^ analogues have also been reported for Te^6+^- and W^6+^-/Mo^6+^-containing double-perovskites, arising from differences in the bonding abilities of the hexavalent cations and resulting in the adoption of different space groups.^69^

At room temperature we find the average structure of Ba_2_GdNbO_6_ to be tetragonal with short-range, *r* < 6 Å, monoclinic distortions. Differences between the local environment and the average crystal structure have been previously observed in perovskites and other systems, including BaTiO_3_, MnO and UO_2_.^42,70,71^ These discrepancies can arise from either local static distortions or dynamic fluctuations. The D4c time scale of measurement, also known as snapshot time, is on the order of femtoseconds, about 100 times shorter than the characteristic time scale of atomic motion, which is approximately 0.1 picoseconds.^71,72^ Therefore, the PDF­(*r*) gives an ensemble-average of quasi-instantaneous local atomic correlations. However, like all diffraction techniques, PDF analysis cannot distinguish between static and dynamic correlations and further measurements would be required to distinguish between the two.^71^

The changes in the structure of Ba_2_GdNbO_6_ also modify the magnetic interactions. As the symmetry is reduced, the *fcc* lattice formed of the Gd^3+^ ions is distorted with four different Gd–Gd distances in the monoclinic polymorph (from 5.973 to 5.996 Å). Likewise, upon symmetry lowering, the Gd–O–Nb–O–Gd superexchange pathways will change. Predicting this *a priori* is complex; however, it can be anticipated that, as *B*/*B′*O_6_ octahedra rotate and the bond angles deviate from 90° and 180°, orbital overlap decreases and the superexchange is weakened. The longer Gd–Gd edge lengths, compared to 5.94 Å reported for Ba_2_GdSbO_6_,^7^ are fully consistent with weaker orbital overlap and reduced superexchange interactions (*J*_1_ = −0.0032(8) K and *J*_1_ = 0.0124(2) K for Ba_2_GdNbO_6_ and Ba_2_GdSbO_6_, respectively). One significant difference in the magnetic properties of the Nb compound when compared to the Sb analogue is the change in sign of the magnetic interactions from ferromagnetic (FM) in Ba_2_GdNbO_6_ (θ = 0.20(5) K) to antiferromagnetic (AFM) in the Sb analogue (θ = −0.78(1) K).^7^ FM correlations are also reported in monoclinic Sr_2_GdNbO_6_,^24^ whereas the monoclinic Sb analogue has AFM correlations.^7^ The change in sign is therefore not only a consequence of the lowered symmetry in Ba_2_GdNbO_6_, but also likely due to the change from filled to empty 4d orbitals when Sb is substituted for Nb. This effect has been investigated extensively for 3d metal ions, including Mn^2+^, Co^2+^, Ni^2+^, and Cu^2+^.^73–76^ In these systems the superexchange interactions are much stronger than those for *Ln* ions, such that dipolar interactions are negligible, and dramatic differences in the magnetic properties are observed on substitution of d^0^ for d^10^ ions on the nonmagnetic *B′*-sites.^73,77^ In transition metal double-perovskites mixing of d^0^ and d^10^ ions on the *B′*-sites has been utilized to tune the magnetic interactions^78,79^ and this might also be possible in the analogous *Ln* systems.

Magnetic measurements confirm the absence of long-range order (LRO) in Ba_2_GdNbO_6_ down to 1.8 K. HC data similarly show no evidence of LRO above this temperature; however, a steep rise in *C*_m_/*T* below 3.74 K suggests the onset of magnetic ordering upon further cooling. We note that Ba_2_GdSbO_6_ remains magnetically disordered down to 0.4 K,^7^ however studies on 5d^1^ double-perovskites indicate that a cubic to tetragonal distortion sometimes correlates with higher magnetic ordering temperatures compared to compounds without such a phase transition.^80^ Lower temperature measurements on Ba_2_GdNbO_6_ are required to see if this correlation holds for *Ln* ^3+^-containing double-perovskites.

FM correlations have previously been invoked as a means to enhance the MCE in lanthanide systems.^81^ However, in the case of Ba_2_Gd*M*O_6_ systems, the Nb compound with FM correlations and the Sb analogue with AFM correlations have comparable MCE, both are high and competitive with other candidate MCE materials. A summary of selected materials is shown in Figure 10 and Table S8, where the MCE per mol_Gd_ is compared to the magnetic ordering temperature or, in cases where no magnetic ordering is observed, the lowest temperature measured. Lower temperature measurements to determine the magnetic behavior of Ba_2_GdNbO_6_ at *T* < 1.8 K are required to further assess its competitiveness as a magnetocaloric material. The possibility for tuning magnetic correlations through doping on the *B′*-site potentially allows for further optimization of the MCE.

**Figure 10:**
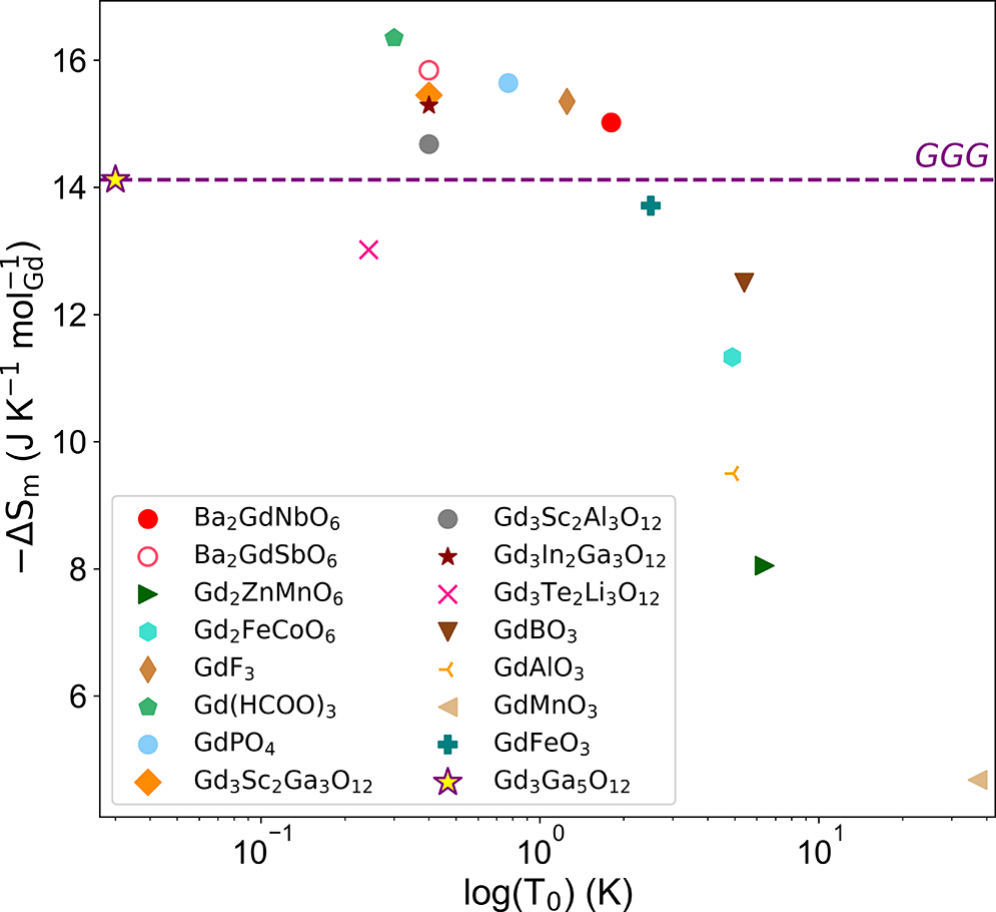
Scatter plot of −Δ*S*_m_ versus magnetic ordering temperature *T*_0_ (or lowest measured temperature, if *T*_0_ is absent) for selected cryogenic materials with large MCE at 5–9 T, comparing the performance of Ba_2_GdNbO_6_ to compounds reported in the literature.^2–7,54,61,82–84^ The −Δ*S*_m_ of the top-performing magnetocaloric material GGG is shown as a dashed line to facilitate comparison.

On the practical side, Ba_2_GdNbO_6_ is a promising material for integration into magnetocaloric cooling devices: it is stable, water-free, avoiding the chemical degradation issues of hydrated salts, and has very weak magnetic interactions, all properties that make it suitable for adiabatic demagnetization refrigeration (ADR) applications.^85^ An estimate of the adiabatic temperature change, Δ*T*_ad_, was computed indirectly from the magnetic entropy change, −Δ*S*_m_ (derived from isothermal *M*(*H*) data), and the zero-field heat capacity, *C*_*p*_ (see details in Section 4 in the Supporting Information).^86^ This yields Δ*T*_ad_ = 21 K at 9 T and 2 K, a large value comparable to the giant MCE reported for GdFeO_3_ (Δ*T*_ad_ = 22 K at 8 T and 5 K).^84^ Future work may include in-field *C*_*p*_ and direct Δ*T*_ad_ measurements to determine definitive quantitative values of Δ*T*_ad_. Despite the presence of several phase transitions in Ba_2_GdNbO_6_, our thermomagnetic data show no magnetic ordering or structural anomalies down to 1.8 K, consistent with very weak spin–lattice coupling, which suggests that the magnetocaloric response of Ba_2_GdNbO_6_ is expected to be reversible and free from significant hysteresis. As a dense oxide with heavy atoms, Ba_2_GdNbO_6_ is expected to be a poor conductor having low thermal conductivity at cryogenic temperatures. This challenge can however be easily overcome by building devices where the magnetocaloric material is mixed with silver powder to enhance its thermal conductivity.^85^

## 4 Conclusions

The structural and magnetic properties of Ba_2_GdNbO_6_ have been comprehensively studied through a combination of PXRD, NPD, neutron PDF, and magnetic measurements. Combined Rietveld refinement of room temperature synchrotron PXRD and NPD data revealed that the compound crystallizes in a tetragonal *I*4/*m* double-perovskite structure with predominant rock-salt ordering of the *B*- and *B′*-sites. On cooling below 100 K, it forms a monoclinic *P*2_1_/*n* structure and on heating, it undergoes a reversible phase transition to a cubic *Fm* 3̅ *m* structure at *T* ≈ 450 K. Neutron PDF analysis confirmed that the average structure exhibits tetragonal symmetry at room temperature but that locally, *r* < 5.9 Å, the structure is best described as monoclinic. A weak SOJT effect is observed at all temperatures (*T* ≤ 300 K) and does not change with temperature.

Magnetically, Ba_2_GdNbO_6_ exhibits FM interactions, with a low Curie–Weiss temperature (θ = 0.20(5) K) and minimal short-range correlations, indicating exceptionally weak spin coupling (*J*_1_ = −3.2(8) mK). The magnetic behavior of Ba_2_GdNbO_6_ is governed by a combination of weak superexchange interactions and dipolar effects, with the tetragonal structure contributing to the reduced spin interactions. This weak magnetic coupling suggests that Ba_2_GdNbO_6_ could be a candidate material for magnetocaloric applications in low-temperature environments.

The magnetic entropy change of the compound (−Δ*S*_m_ = 15.75 J K^–1^ mol^–1^ at 2 K and 9 T) is comparable to that of the Ba_2_GdSbO_6_ analogue and exceeds that of GGG in molar units, indicating promising magnetocaloric properties. The high Debye temperature (*T*_D_ = 267(3) K) computed from temperature-dependent HC measurements indicates a rigid crystal lattice and a corresponding small lattice entropy in the low-temperature regime, supporting the suitability of Ba_2_GdNbO_6_ for magnetocaloric applications and its potential for cryogenic cooling applications.

## Supplementary Material



## Data Availability

Neutron data associated with the ILL beamtime are available at https://doi.org/10.5291/ILL-DATA.5-23-807.^87^ All other data are available in the University of Cambridge repository at https://doi.org/10.17863/CAM.120099.^88^

## Notes

Additional structural characterization, including quantification of SOJT distortion; isothermal magnetization; comparison of the MCE in cryogenic materials; estimate of the adiabatic temperature change; bond valence sum analysis; and magnetocaloric cooling rate (PDF)

## ∥

Stanford Synchrotron Radiation Lightsource, SLAC National Accelerator Laboratory, Menlo Park, CA 94025, United States of America
