# Supplementary material for: Structural Phase Transitions and Magnetic Characterization of Ba2GdNbO6 for Low-Temperature Magnetocaloric Refrigeration
Source: Chem Mater. 2025 Oct 27;37(21):8848–60. doi: 10.1021/acs.chemmater.5c01937 (PMC12613325; doi:10.1021/acs.chemmater.5c01937)
Supplement: Supplementary file 1 [file cm5c01937_si_001.pdf]

Supporting Information: Structural Phase Transitions and Magnetic Characterization of Ba_2GdNbO_6 for Low-Temperature Magnetocaloric Refrigeration

Fiamma Berardi,[1*] Liam A. V. Nagle-Cocco,[1,2] James M. A. Steele,[3,1] Xiaotian Zhang,[1] Cheng Liu,[1] Henry E. Fischer,[4] Siân E. Dutton[1*]

[*] Corresponding authors: fb560@cam.ac.uk; sed33@cam.ac.uk

[1] Cavendish Laboratory, University of Cambridge, JJ Thomson Avenue, Cambridge CB3 0US, United Kingdom

[2] Present Address: Stanford Synchrotron Radiation Lightsource, SLAC National Accelerator Laboratory, Menlo Park, CA 94025, United States of America

[3] Yusuf Hamied Department of Chemistry, University of Cambridge, Cambridge CB2 1EW, United Kingdom

[4] Institut Laue-Langevin, 71 Avenue des Martyrs, CS 20156, 38042 Grenoble cedex 9, France

Table of Contents

1. STRUCTURAL CHARACTERIZATION 2

1.1. Rietveld refinements of room temperature structure 2

1.2. Monoclinic distortion metric from box-car PDF analysis 4

1.3. Variable-temperature NPD data 5

1.4. Variable-temperature PXRD data 6

1.5. Rietveld refinement of NPD data at 2.4 K 9

1.6. Quantification of second-order Jahn-Teller distortion 10

2. ISOTHERMAL MAGNETIZATION 12

3. COMPARISON OF THE MCE IN CRYOGENIC MATERIALS 13

4. ESTIMATE OF THE ADIABATIC TEMPERATURE CHANGE 14

5. BOND VALENCE SUM ANALYSIS 15

6. MAGNETOCALORIC COOLING RATE 16

REFERENCES 18

1. STRUCTURAL CHARACTERIZATION

1.1. Rietveld refinements of room temperature structure

Table S1. Room temperature Rietveld refinement results for all reported space groups for Ba_2GdNbO_6. For the refinement in the $Pm\bar{3}m$ space group, the structure was refined as $Ba(Gd_{0.5}Nb_{0.5})O_3$, as in the literature, with Gd^{3+} and Nb^{5+} occupying the *B*-site with equal 0.5 occupancy. Fitting metrics R_{wp} and χ^2 have been rounded to the second decimal place.

Space group	$I4/m$	$I4/m$	$P2_1/n$	$Fm\bar{3}m$	$Pm\bar{3}m$
Dataset	PXRD + NPD	PXRD	PXRD	PXRD	PXRD
a (Å)	5.996284(8)	5.99611(2)	5.99610(16)	8.4896(4)	4.24457(18)
b (Å)	5.996284(8)	5.99611(2)	5.99615(15)	8.4896(4)	4.24457(18)
c (Å)	8.515386(18)	8.51543(3)	8.51543(3)	8.4896(4)	4.24457(18)
β (°)	90	90	90.0272(5)	90	90
V (Å^3)	306.1743(15)	306.1585(19)	306.160(11)	611.88(8)	76.472(10)
V/Z (Å^3/*f.u.*)	76.5436(4)	76.5396(5)	76.540(3)	76.485(10)	76.472(10)
Ba					
Wyckoff	4*d*	4*d*	4*e*	8*c*	1*a*
x	0	0	-0.0004(10)	0.25	0
y	0.5	0.5	0.5003(6)	0.25	0
z	0.25	0.25	0.7460(2)	0.25	0
B_{iso} (Å^2)	1.67(4)	0.78(2)	0.75(2)	0.84(13)	1.04(4)
Gd					
Wyckoff	2*a*	2*a*	2*a*	4*b*	1*b*
x	0	0	0	0.5	0.5
y	0	0	0	0.5	0.5
z	0	0	0	0.5	0.5
B_{iso} (Å^2)	0.13(3)	0.494(18)	0.56(2)	0.61(12)	0.10(7)
Nb					
Wyckoff	2*b*	2*b*	2*c*	4*a*	1*b*
x	0	0	0	0	0.5
y	0	0	0	0	0.5
z	0.5	0.5	0.5	0	0.5
B_{iso} (Å^2)	0.37(5)	0.41(3)	0.30(3)	1.1(2)	16.0(9)
O1					
Wyckoff	4*e*	4*e*	4*e*	24*e*	3*c*
x	0	0	-0.009(5)	0.225(2)	0.5
y	0	0	-0.030(4)	0	0.5
z	0.2689(4)	0.2674(8)	0.2677(8)	0	0
B_{iso} (Å^2)	0.43(4)	1.67(13)	0.7(2)	0.56(2)	19.0(9)
O2					
Wyckoff	8*h*	8*h*	4*e*		
x	0.2392(7)	0.2473(12)	0.244(7)		
y	0.2926(7)	0.2902(11)	0.705(7)		
z	0	0	0.012(4)		
B_{iso} (Å^2)	4.08(10)	1.35(11)	1.48(8)		
O3					
Wyckoff			4*e*		
x			0.278(8)		
y			0.258(7)		
z			-0.008(4)		
B_{iso} (Å^2)			1.48(8)		
R_{wp} (%)	4.66	3.96	3.88	20.33	20.04
χ^2	5.62	4.51	4.42	23.15	22.81

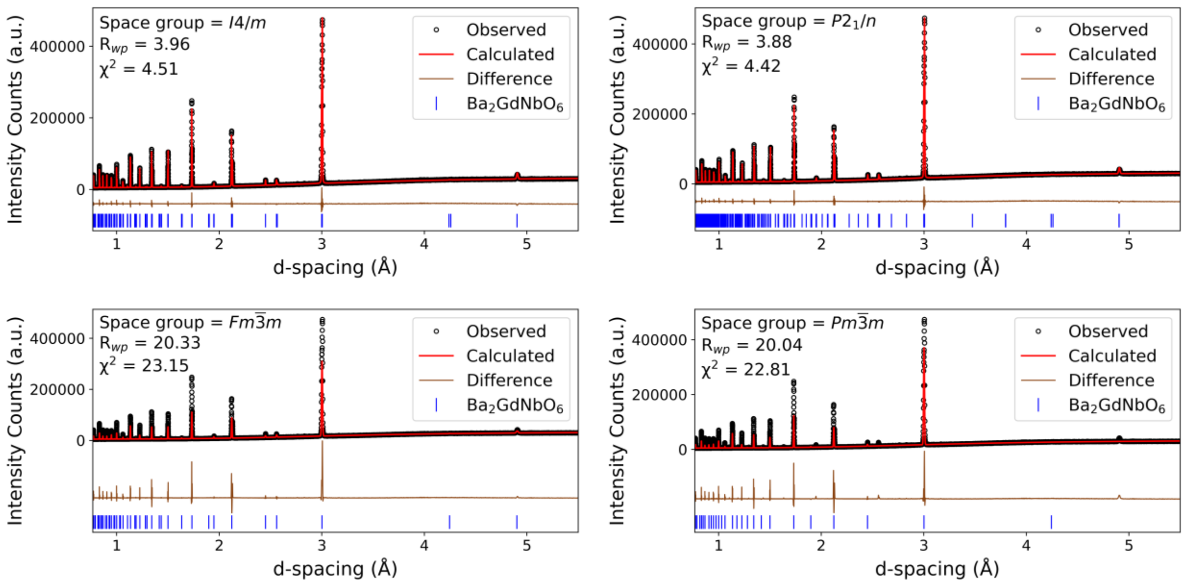

Figure S1. Rietveld refinements of room temperature synchrotron PXRD in the space groups reported in the literature. Open black circles correspond to experimental data points, the red line to the calculated Rietveld fit, the brown line to the difference between the two, and the blue tick marks to Bragg reflection positions.

1.2. Monoclinic distortion metric from box-car PDF analysis

Due to the modest r-space resolution of 0.16 Å of the D4c diffractometer, group-subgroup relation between $I4/m$ and $P2_1/n$ and more degrees of freedom, the monoclinic symmetry fits better than the tetragonal symmetry over all r-ranges when performing a box-car analysis of the room temperature neutron PDF data. This method involves taking fixed-length boxes and performing sequential refinements from low to high r-ranges.[1] Here, a fixed refinement range of 6 Å was used to refine the structure from 1 to 40 Å (over prescribed ranges of 1–6 Å, 6–12 Å, etc., except for the last box, which was 36–40 Å). Although the monoclinic symmetry fits better than the tetragonal symmetry, a monoclinic distortion metric was defined to show that the lattice parameters of the monoclinic cell essentially converge to tetragonal symmetry at longer length scales, supporting our argument of an average tetragonal structure with local monoclinic distortions (r < 6 Å). The monoclinic distortion metric was expressed as the ratio of the two lattice parameters of the refined monoclinic cell, $R_{ab}=a_{mon}/b_{mon}$. By construction, $R_{ab}=1$ for a perfectly tetragonal lattice ($a=b$). When the ratio deviates from one, the degree of monoclinic distortion in the *ab* plane is quantified. The gradual increase in the calculated ratio, from ~0.965 at 3.5 Å toward 1 at 38 Å, indicates that at high-r the structure is most appropriately described as tetragonal.

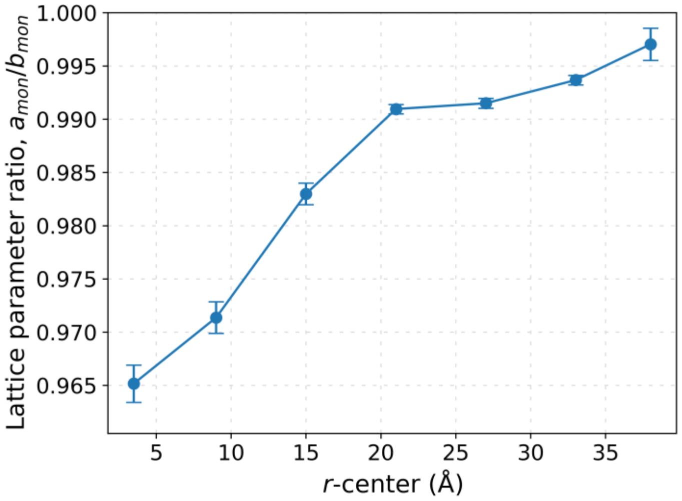

Figure S2. Monoclinic lattice parameter ratio, a_{mon}/b_{mon}, versus the r-center used in the box-car neutron PDF analysis of Ba_2GdNbO_6 at 300 K. The box width is 6 Å, and the x-axis values correspond to the midpoint of each box.

1.3. Variable-temperature NPD data

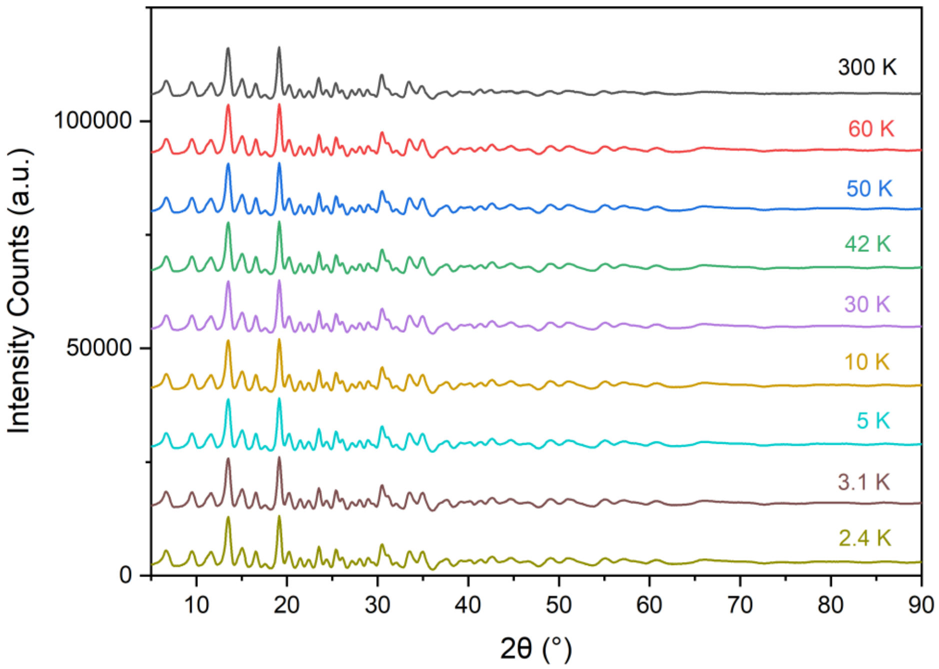

Figure S3. NPD patterns collected at variable temperature on cooling (λ = 0.4982 Å).

1.4. Variable-temperature PXRD data

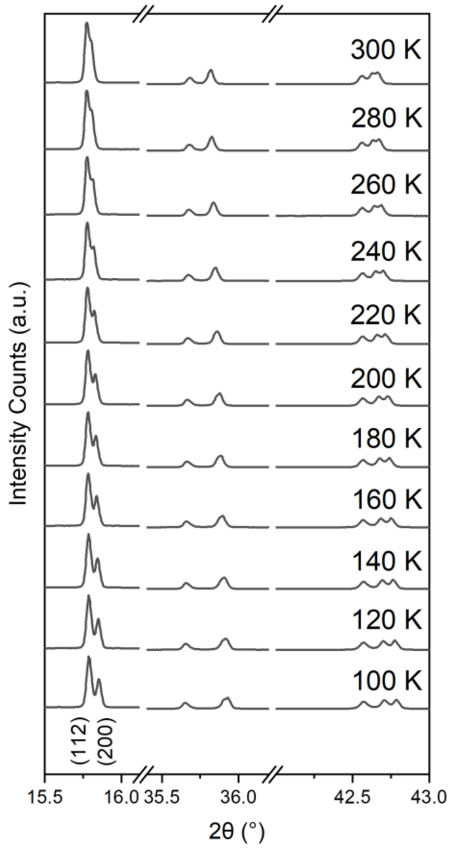

Figure S4. Zoomed-in regions of synchrotron PXRD data, highlighting peak splitting of selected reflections upon cooling (λ = 0.82483 Å).

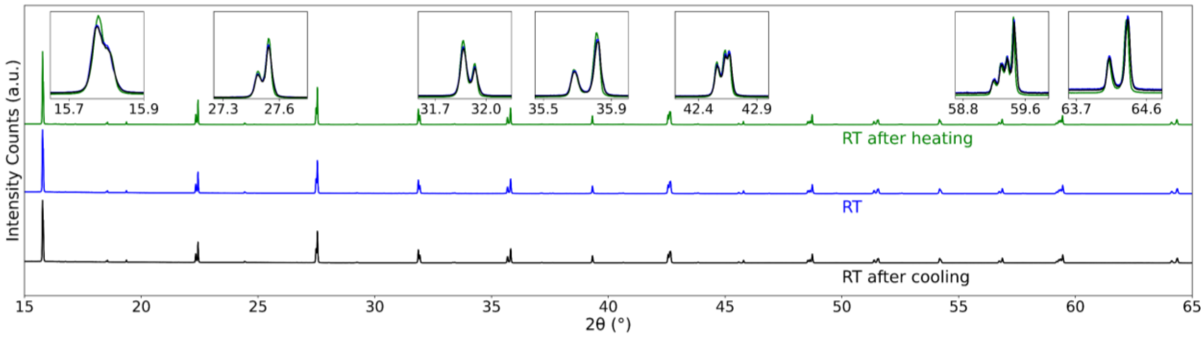

Figure S5. Synchrotron VT-PXRD data showing the reversibility of the phase transitions in Ba_2GdNbO_6. The blue line shows room temperature (RT) data collected before VT measurements, the green line shows RT data collected after heating to 950 K, and the black line shows RT data collected after cooling to 100 K. Zoomed in regions are included to highlight the reversibility of peak merging and splitting.

Table S2. Refined crystallographic data for Ba_2GdNbO_6 obtained from sequential Rietveld refinement of synchrotron PXRD data [space group: $I4/m$; O1 (4*e*: 0, 0, *z*); O2 (8*h*: *x*, *y*, 0)] at selected temperatures. Fitting metrics R_{wp} and χ^2 have been rounded to the second decimal place.

T (K)	100	150	200	250	300	445
a, b (Å)	5.97894(2)	5.98290(2)	5.98681(2)	5.99203(2)	5.99611(2)	6.012860(11)
c (Å)	8.52586(4)	8.52315(3)	8.52064(3)	8.51752(3)	8.51543(3)	8.50708(3)
V (Å^3)	304.780(3)	305.0874(19)	305.3955(18)	305.817(2)	306.1585(19)	307.5692(15)
V/Z (Å^3/*f.u.*)	76.195(1)	76.2718(5)	76.3489(4)	76.4543(5)	76.5396(5)	76.8923(4)
$Dist_t$ (%)	0.8287(6)	0.7307(8)	0.6359(8)	0.5122(8)	0.4196(8)	0.0424(7)
Ba (4*d*)						
(0, ½, ¼)						
B_{iso} (Å^2)	0.37(2)	0.45(2)	0.54(2)	0.69(2)	0.78(2)	1.148(11)
Gd (2*a*)						
(0, 0, 0)						
B_{iso} (Å^2)	0.43(2)	0.43(2)	0.44(2)	0.459(19)	0.494(18)	0.753(12)
Nb (2*b*)						
(0, 0, ½)						
B_{iso} (Å^2)	0.44(4)	0.48(4)	0.45(4)	0.39(3)	0.41(3)	0.515(16)
O1 (4*e*)						
z	0.2652(9)	0.2654(9)	0.2655(8)	0.2663(8)	0.2674(8)	0.272(2)
B_{iso} (Å^2)	0.97(12)	1.03(12)	1.05(12)	0.99(12)	1.67(13)	5.2(5)
O2 (8*h*)						
x	0.2990(10)	0.2388(10)	0.2955(11)	0.2416(10)	0.2473(12)	0.2875(13)
y	0.2370(10)	0.2991(10)	0.2444(11)	0.2932(10)	0.2902(11)	0.2420(14)
B_{iso} (Å^2)	0.68(12)	0.78(12)	1.12(12)	0.99(11)	1.35(11)	0.01(14)
R_{wp} (%)	5.52	5.23	5.04	4.80	3.96	5.62
χ^2	5.91	5.70	5.42	5.17	4.51	6.85

Table S3. Refined crystallographic data for Ba_2GdNbO_6 obtained from sequential Rietveld refinement of synchrotron PXRD data [space group: $Fm\bar{3}m$; O1 (24*e*: *x*, 0, 0)] at selected temperatures. Fitting metrics R_{wp} and χ^2 have been rounded to the second decimal place.

T (K)	460	555	655	755	855	950
a, b, c (Å)	8.50423(1)	8.51098(1)	8.51790(1)	8.52442(1)	8.53113(1)	8.53748(1)
V (Å^3)	615.043(3)	616.509(3)	618.013(2)	619.433(2)	620.898(2)	622.285(2)
V/Z (Å^3/*f.u.*)	76.8803(4)	77.0636(3)	77.2516(3)	77.4292(3)	77.6122(3)	77.7856(3)
Ba (8*c*)						
(¼, ¼, ¼)						
B_{iso} (Å^2)	0.674(7)	0.481(8)	0.276(10)	0.106(13)	0.214(16)	0.491(14)
Gd (4*b*)						
(½, ½, ½)						
B_{iso} (Å^2)	0.001(12)	0.001(11)	0.001(11)	0.001(13)	0.001(14)	0.0001(118)
Nb (4*a*)						
(0, 0, 0)						
B_{iso} (Å^2)	0.01(2)	0.001(19)	0.01(2)	0.01(2)	0.01(2)	0.0001(206)
O1 (24*e*)						
x	0.7316(5)	0.7300(4)	0.7313(4)	0.7319(4)	0.7322(3)	0.7313(3)
B_{iso} (Å^2)	1.64(8)	1.56(7)	0.99(6)	1.22(6)	1.02(6)	1.08(6)
R_{wp} (%)	7.86	6.81	6.01	6.02	5.66	5.75
χ^2	9.56	8.29	7.29	7.28	6.81	6.93

Table S4. Linear thermal expansion coefficients, α, along the three principal axes of the tetragonal unit cell, determined using PASCal (Principal Axis Strain Calculator).[2] The corresponding directions are given in terms of the crystallographic axes (*a*, *b*, *c*). The volumetric expansion coefficient, V, is also shown.

Axis	α (MK^{-1})	σα (MK^{-1})	Direction (*a*, *b*, *c*)
X_1	-6.3789	0.053	(0.0, 0.0, 1.0)
X_2	16.3975	0.6733	(1.0, 0.0, 0.0)
X_3	16.3975	0.6733	(0.0, 1.0, 0.0)
V	26.5363	1.3271	

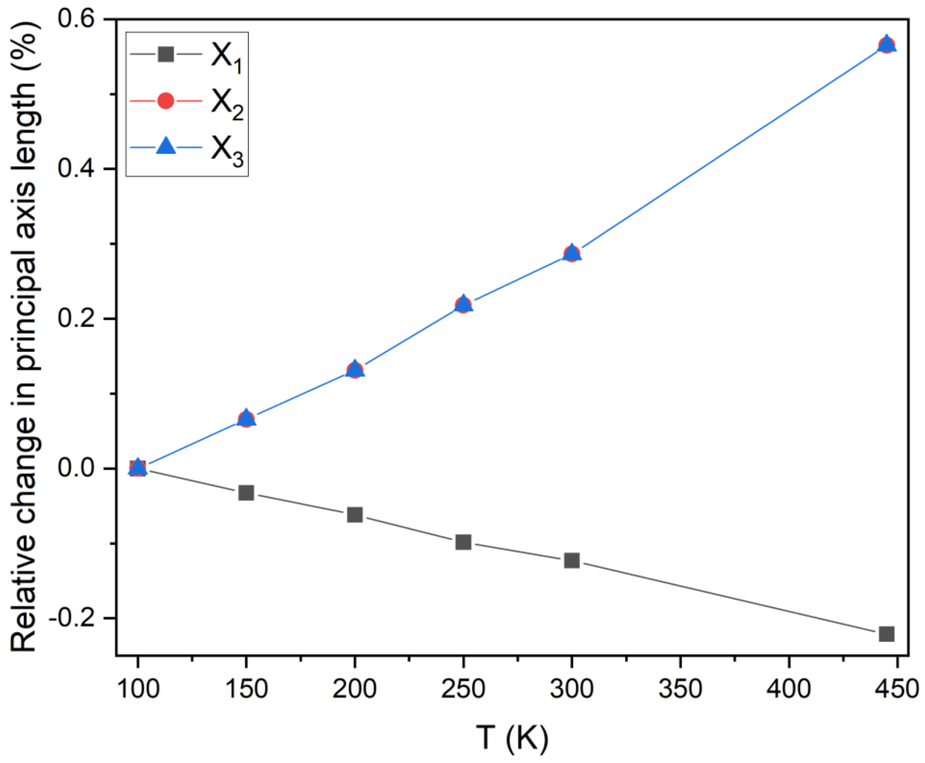

Figure S6. Percent length change of the three principal axes of the tetragonal unit cell versus temperature, as calculated from the PASCal output. X_1 corresponds to the *c* axis, X_2 to the *a* axis, and X_3 to the *b* axis, as described in Table S4. Axis X_1 exhibits negative thermal expansion (α < 0).

1.5. Rietveld refinement of NPD data at 2.4 K

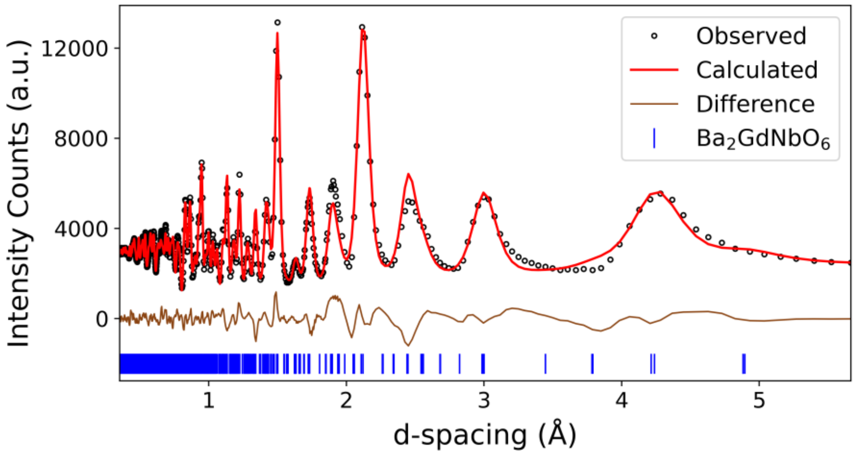

Figure S7. Rietveld refinement of NPD at 2.4 K. Open black circles correspond to experimental data points, the red line to the calculated Rietveld fit, the brown line to the difference between the two, and the blue tick marks to Bragg reflection positions.

Table S5. Structural parameters and Wyckoff positions of Ba_2GdNbO_6 obtained from Rietveld refinement of NPD at 2.4 K in the space group $P2_1/n$.

R_{wp} = 6.51%; χ^2 = 13.27					
a = 5.996(6) Å, b = 5.988(7) Å, c = 8.435(7) Å, β = 90.14(9)°, volume = 302.9(5) Å^3					
atom	Wyckoff position	x	y	z	B_{iso} (Å^2)
Ba	4e	-0.008(2)	0.484(4)	0.741(3)	0.5(2)
Gd	2a	0	0	0	0.06(10)
Nb	2c	0	0	0.5	0.17(8)
O(1)	4e	0.273(3)	0.264(3)	-0.0188(14)	0.30(15)
O(2)	4e	0.449(9)	0.452(11)	0.233(9)	13.9(18)
O(3)	4e	0.240(3)	0.231(4)	0.5300(15)	0.44(13)

1.6. Quantification of second-order Jahn-Teller distortion

The magnitude of the SOJT distortion, Δ_d, was calculated as follows:[3]

$$\Delta_d = \left(\frac{|(Nb-O1)-(Nb-O4)|}{|\cos\theta_1|}\right) + \left(\frac{|(Nb-O2)-(Nb-O5)|}{|\cos\theta_2|}\right) + \left(\frac{|(Nb-O3)-(Nb-O6)|}{|\cos\theta_3|}\right) \quad \text{(S1)}$$

where $Nb-O$ stands for the six bond lengths, and θ stands for the three *trans* $O-Nb-O$ bond angles. Following the numbering scheme given in Figure S8, $\theta_1 = \measuredangle O1-Nb-O4$, $\theta_2 = \measuredangle O2-Nb-O5$, and $\theta_3 = \measuredangle O3-Nb-O6$. Values for Δ_d and for the off-centering distance d_{oct} as a function of temperature are shown in Figure S9 and Table S6.

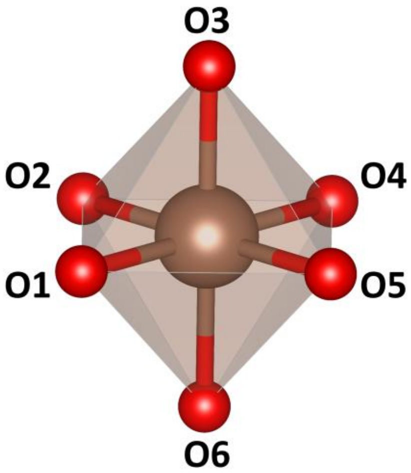

Figure S8. NbO_6 octahedron with numbered oxygen atoms to identify *trans* bond lengths.

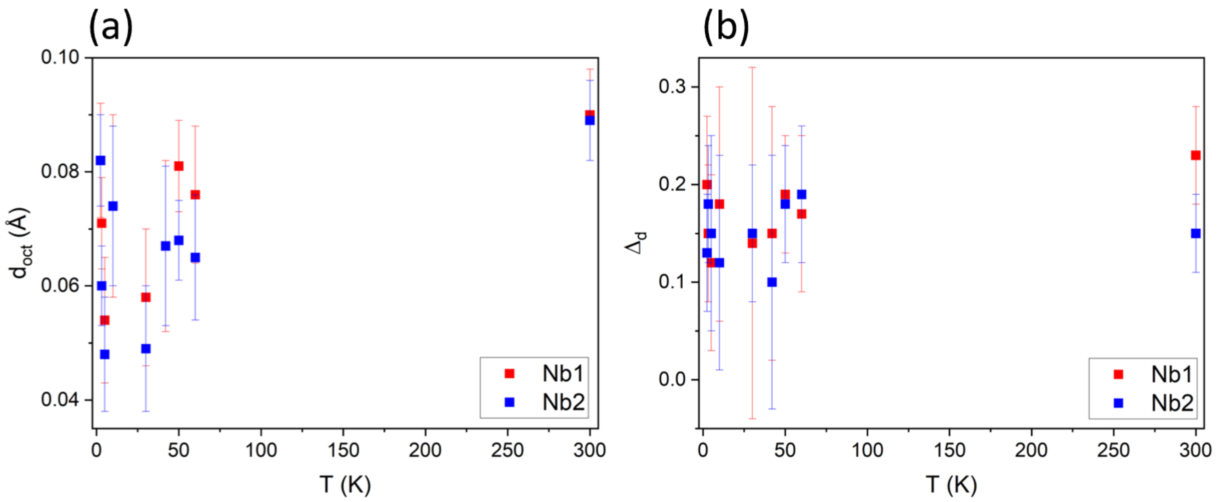

Figure S9. (a) d_{oct} and (b) Δ_d for the Nb1 and Nb2 sites plotted against temperature to show the temperature invariance of the off-centering parameters.

Table S6. Calculated off-centering values, d_{oct} and Δ_d (as defined in Equations 7 and S1, respectively), for the Nb1 and Nb2 sites at all measured temperatures, as calculated by small-box analysis of the neutron PDF data.

	d_{oct} (Å)	Δ_d
300 K		
Nb1	0.090(8)	0.23(5)
Nb2	0.089(7)	0.15(4)
60 K		
Nb1	0.076(12)	0.17(8)
Nb2	0.065(11)	0.19(7)
50 K		
Nb1	0.081(8)	0.19(6)
Nb2	0.068(7)	0.18(6)
42 K		
Nb1	0.067(15)	0.15(13)
Nb2	0.067(14)	0.10(13)
30 K		
Nb1	0.058(12)	0.14(18)
Nb2	0.049(11)	0.15(7)
10 K		
Nb1	0.074(16)	0.18(12)
Nb2	0.074(14)	0.12(11)
5 K		
Nb1	0.054(11)	0.12(9)
Nb2	0.048(10)	0.15(10)
3.1 K		
Nb1	0.071(8)	0.15(7)
Nb2	0.060(7)	0.18(6)
2.4 K		
Nb1	0.082(10)	0.20(7)
Nb2	0.082(8)	0.13(6)

2. ISOTHERMAL MAGNETIZATION

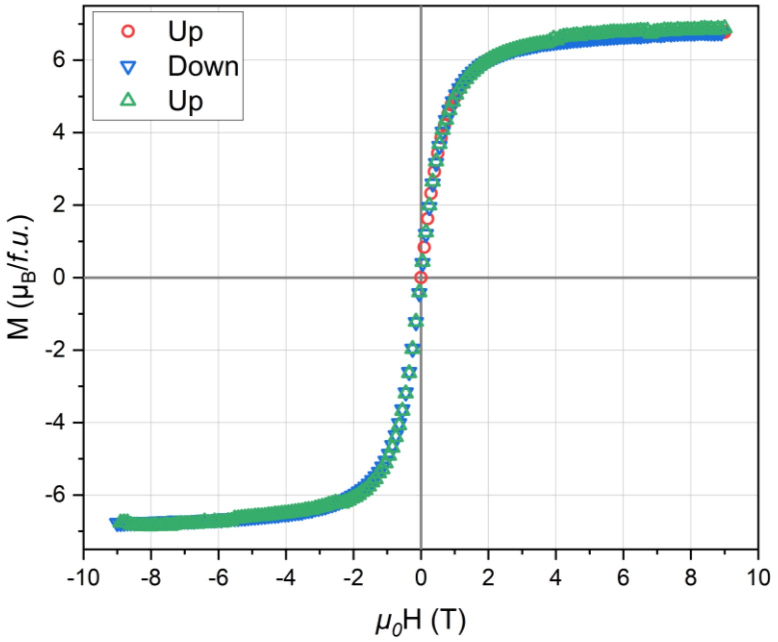

Figure S10. Five-loop (0 T → 9 T → -9 T → 9 T) isothermal magnetization measured at 2 K showing no hysteresis.

3. COMPARISON OF THE MCE IN CRYOGENIC MATERIALS

Table S7. Comparison of the magnetic entropy change for Ba_2GdNbO_6 and Ba_2GdSbO_6 expressed in molar, gravimetric, and volumetric units.

Material	$-\Delta S_m$ (J K^{-1} mol^{-1})	$-\Delta S_m$ (J K^{-1} kg^{-1})	$-\Delta S_m$ (mJ K^{-1} cm^{-3})
**Ba_2GdNbO_6**	15.75	25.37	170.84
Ba_2GdSbO_6[4]	15.84	24.38	172.86

Table S8. Magnetic entropy change of selected cryogenic materials with large magnetocaloric effect at 5–9 T, comparing the performance of Ba_2GdNbO_6 to compounds reported in the literature.

Material	$-\Delta S_m$ (J K^{-1} mol_{Gd}^{-1})	ΔH (T)	T (K)	Magnetic ordering temperature $T_0 \leq$ (K)
**Ba_2GdNbO_6**	15.75	9	2	1.8
Ba_2GdSbO_6[4]	15.84	7	2	0.4
Gd_2ZnMnO_6[5]	8.05	5	2	6.4
Gd_2FeCoO_6[6]	11.33	5	2	4.9
GdF_3[7]	15.35	7	2	1.25
$Gd(HCOO)_3$[8]	16.35	7	2	0.3
$GdPO_4$[9]	15.64	7	2	0.77
$Gd_3Sc_2Ga_3O_{12}$[10]	15.45	9	2	0.4
$Gd_3Sc_2Al_3O_{12}$[10]	14.68	9	2	0.4
$Gd_3In_2Ga_3O_{12}$[10]	15.29	9	2	0.4
$Gd_3Te_2Li_3O_{12}$[10]	13.02	9	2	0.243
$GdBO_3$[11]	12.5	9	2	5.4
$GdAlO_3$[12]	9.50	9	2	4.95
$GdMnO_3$[12]	4.68	9	2	37.15
$GdFeO_3$[13]	13.71	9	2	2.5
$Gd_3Ga_5O_{12}$[10, 14]	14.12	9	2	0.03

4. ESTIMATE OF THE ADIABATIC TEMPERATURE CHANGE

The adiabatic temperature change, ΔT_{ad}, was estimated indirectly from the magnetic entropy change, $-\Delta S_{\mathrm{m}}$ (derived from isothermal $M(H)$ data), and the zero-field heat capacity, C_{p} (after subtraction of the silver contribution). For the numerical treatment, both the measured C_{p} and the $-\Delta S_{\mathrm{m}}$ points were interpolated on a fine temperature grid using the Piecewise Cubic Hermite Interpolating Polynomial (PCHIP). PCHIP is a shape-preserving method that maintains monotonicity of the data and avoids oscillations and overshoots. Entropies were then obtained by numerical integration of C_{p}/T and roots were found using Brent's method to determine the adiabatic temperature shift (i.e. solving for the temperature change that keeps entropy constant). The following widely reported approximate relation was implemented as a fallback method, but it was not required in practice:[15]

$$\Delta T_{\mathrm{ad}} \approx -\frac{T\Delta S_{\mathrm{m}}}{C_{\mathrm{p}}} \tag{S2}$$

Figure S11 shows the indirect estimate of the adiabatic temperature change ΔT_{ad} and the magnetic entropy change $-\Delta S_{\mathrm{m}}$ for ΔH = 9 T. The approach used assumes negligible field dependence of C_{p} (justified by the absence of magnetic transitions in the temperature range investigated) and reversibility of the magnetization; therefore, the reported ΔT_{ad} should be considered an estimate. Direct ΔT_{ad} measurements and in-field C_{p} would be needed for precise quantitative values.

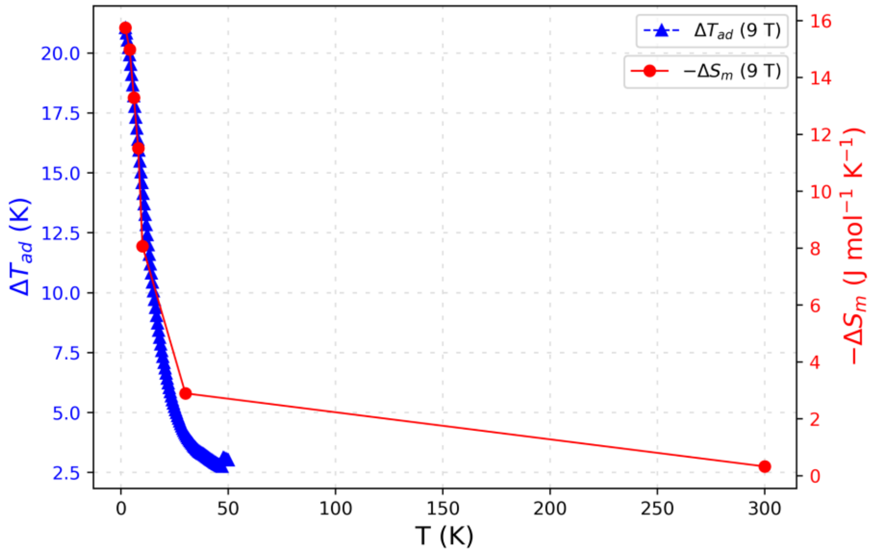

Figure S11. Indirect estimate of the adiabatic temperature change ΔT_{ad} (left axis) and the magnetic entropy change $-\Delta S_{\mathrm{m}}$ (right axis) for ΔH = 9 T.

5. BOND VALENCE SUM ANALYSIS

Bond valence sum (BVS) analysis was performed to obtain the oxidation states of the cations in Ba_2GdNbO_6. Values were calculated using the equation defined in Brown and Altermatt, *Acta Cryst.* **B41**, 244–247. The bond length R_i was taken from the room temperature combined XRD and NPD Rietveld refinement, R_0 is the tabulated ideal bond length, and *B* is an empirical constant.[16, 17] Table S9 shows the calculated BVS value for each cation, indicating that all the cations are in their expected oxidation state (Ba^{2+}, Gd^{3+}, Nb^{5+}).

Table S9. Bond valence sum (BVS) values for the cations in Ba_2GdNbO_6.

Cation	Anion	R_i (Å)	R_0 (Å)	*B*	$\exp[(R_0–R_i)/B]$	Multiplicity	BVS	Ox. state
	O2	2.852	2.223	0.406	0.212404865	4		
Ba	O1	3.002	2.223	0.406	0.146650301	4	1.82252	2+
	O2	3.172	2.223	0.406	0.096574705	4		
Gd	O2	2.266	1.988	0.433	0.526221879	4	3.10289	3+
	O1	2.289	1.988	0.433	0.498999535	2		
Nb	O1	1.968	1.916	0.37	0.868888439	2	4.94265	5+
	O2	1.998	1.916	0.37	0.801218471	4		

6. MAGNETOCALORIC COOLING RATE

The magnetocaloric cooling rate is a valuable parameter to determine the saturation field H_{sat}, since an enhanced MCE near H_{sat} for geometrically frustrated magnets was predicted by Zhitomirsky and then by Sosin *et al.*.[18, 19] The degeneracy of the magnetic ground state of frustrated materials in zero-field arises from the impossibility to satisfy all microscopic degrees of freedom. The spins align with the applied field when $H > H_{sat}$, forming a unique, non-degenerate and fully polarised ground state, which becomes infinitely degenerate when $H < H_{sat}$. This transition occurs at $H = H_{sat}$ via the condensation of a macroscopic number of zero-energy modes (also called "soft modes"), which are excitations arising from the degeneracy. Therefore, at $H = H_{sat}$, frustrated magnets have a macroscopic entropy, which does not depend on the value of the spin of the magnetic ions but comes from the condensation of the soft modes. Zhitomirsky predicts that a large number of soft modes enhances the MCE and the proportionality at $H = H_{sat}$ is given by:[18]

$$\left(\frac{\partial S}{\partial H}\right)_T \propto -\frac{N_4}{\sqrt{J_1 T}} \tag{S3}$$

Where: S is entropy, H is magnetic field, T is temperature, N_4 is the number of soft modes and J_1 is the *nn* exchange

The cooling rate is maximized for materials with large negative values of $\left(\frac{\partial S}{\partial H}\right)_T$. The magnetocaloric cooling rate for the Ba_2GdNbO_6 at 2 K was determined experimentally by applying one of Maxwell's relations:

$$\left(\frac{\partial S}{\partial H}\right)_T = \left(\frac{\partial M}{\partial T}\right)_H \tag{S4}$$

Plotting $-(\Delta M/\Delta T)_H \approx -(\partial M/\partial T)_H = -(\partial S/\partial H)_T$ versus field, $\mu_0 H$, the saturation field, H_{sat}, was estimated from the field at which the temperature gradient of the magnetization is maximized (Figure S12).[20] The value of H_{sat} obtained (0.72 T) is in line with that reported for the Ba_2GdSbO_6 analogue (1.4 T).[20] As for Ba_2GdSbO_6, this result suggests that Ba_2GdNbO_6 is described quantitatively well in the paramagnetic regime by the mean-field *nn* superexchange model across the full range of temperatures of interest in this study. Therefore, the paramagnetic response dominates and is expected to be the main contribution to the measured magnetic entropy change, $-\Delta S_{\mathrm{m}}$.[20]

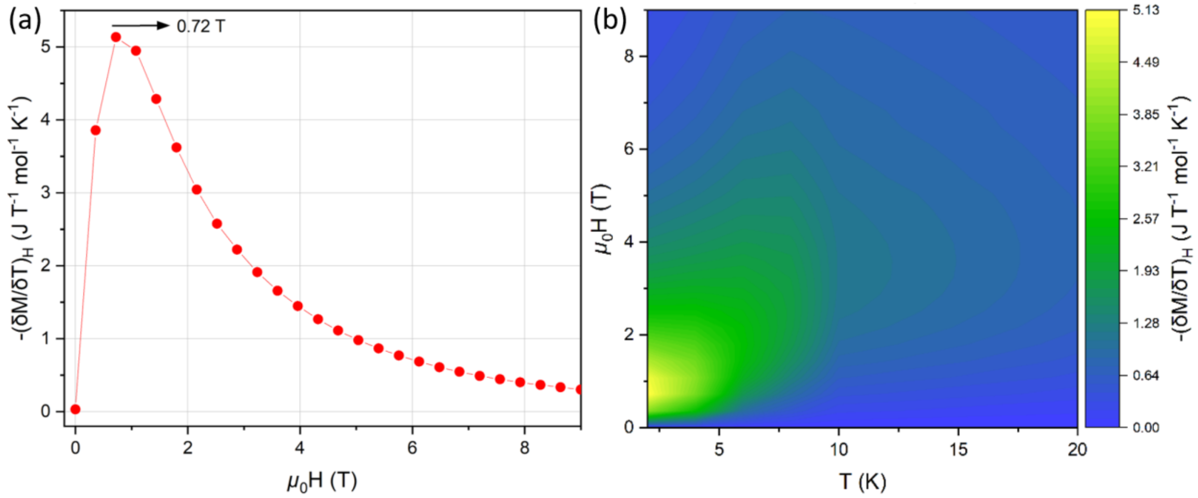

Figure S12. (a) Approximate magnetocaloric cooling rate for Ba_2GdNbO_6, extracted from the measured isothermal magnetization at $T = 2\ K$. The resulting value of H_{sat} is indicated by the arrow. (b) Heatmap of the magnetocaloric cooling rate, as a function of T and $\mu_0 H$.

REFERENCES

(1) Culbertson, C. M.; Flak, A. T.; Yatskin, M.; Cheong, P. H. Y.; Cann, D. P.; Dolgos, M. R. Neutron Total Scattering Studies of Group II Titanates ($ATiO_3$, A^{2+} = Mg, Ca, Sr, Ba). *Scientific Reports* **2020**, *10* (1), 3729. DOI: https://doi.org/10.1038/s41598-020-60475-8.

(2) Monthakan Lertkiattrakul, M. L. E., Matthew J. Cliffe. PASCal Python: A Principal Axis Strain Calculator. *Journal of Open Source Software* **2023**, *8* (90), 5556. DOI: https://doi.org/10.21105/joss.05556.

(3) Halasyamani, P. S. Asymmetric Cation Coordination in Oxide Materials: Influence of Lone-Pair Cations on the Intra-octahedral Distortion in d^0 Transition Metals. *Chemistry of Materials* **2004**, *16* (19), 3586-3592. DOI: https://doi.org/10.1021/cm049297g.

(4) Koskelo, E. C.; Liu, C.; Mukherjee, P.; Kelly, N. D.; Dutton, S. E. Free-Spin Dominated Magnetocaloric Effect in Dense Gd^{3+} Double Perovskites. *Chemistry of Materials* **2022**, *34* (7), 3440-3450. DOI: https://doi.org/10.1021/acs.chemmater.2c00261.

(5) Li, L.; Xu, P.; Ye, S.; Li, Y.; Liu, G.; Huo, D.; Yan, M. Magnetic properties and excellent cryogenic magnetocaloric performances in *B*-site ordered RE_2ZnMnO_6 (*RE* = Gd, Dy and Ho) perovskites. *Acta Materialia* **2020**, *194*, 354-365. DOI: https://doi.org/10.1016/j.actamat.2020.05.036.

(6) Dong, Z.; Yin, S. Structural, magnetic and magnetocaloric properties in perovskite RE_2FeCoO_6 (*RE* = Er and Gd) compounds. *Ceramics International* **2020**, *46* (1), 1099-1103. DOI: https://doi.org/10.1016/j.ceramint.2019.09.077.

(7) Chen, Y.-C.; Prokleška, J.; Xu, W.-J.; Liu, J.-L.; Liu, J.; Zhang, W.-X.; Jia, J.-H.; Sechovský, V.; Tong, M.-L. A brilliant cryogenic magnetic coolant: magnetic and magnetocaloric study of ferromagnetically coupled GdF_3. *Journal of Materials Chemistry C* **2015**, *3* (47), 12206-12211. DOI: https://doi.org/10.1039/C5TC02352A.

(8) Lorusso, G.; Sharples, J. W.; Palacios, E.; Roubeau, O.; Brechin, E. K.; Sessoli, R.; Rossin, A.; Tuna, F.; McInnes, E. J. L.; Collison, D.; et al. A Dense Metal–Organic Framework for Enhanced Magnetic Refrigeration. *Advanced Materials* **2013**, *25* (33), 4653-4656. DOI: https://doi.org/10.1002/adma.201301997.

(9) Palacios, E.; Rodríguez-Velamazán, J. A.; Evangelisti, M.; McIntyre, G. J.; Lorusso, G.; Visser, D.; de Jongh, L. J.; Boatner, L. A. Magnetic structure and magnetocalorics of $GdPO_4$. *Phys Rev B* **2014**, *90* (21), 214423. DOI: https://doi.org/10.1103/PhysRevB.90.214423.

(10) Mukherjee, P.; Sackville Hamilton, A. C.; Glass, H. F. J.; Dutton, S. E. Sensitivity of magnetic properties to chemical pressure in lanthanide garnets $Ln_3A_2X_3O_{12}$, *Ln* = Gd, Tb, Dy, Ho, *A* = Ga, Sc, In, Te, *X* = Ga, Al, Li. *Journal of Physics Condensed Matter* **2017**, *29* (40), 405808. DOI: https://doi.org/10.1088/1361-648X/aa810e.

(11) Mukherjee, P.; Wu, Y.; Lampronti, G. I.; Dutton, S. E. Magnetic properties of monoclinic lanthanide orthoborates, $LnBO_3$, *Ln*=Gd, Tb, Dy, Ho, Er, Yb. *Materials Research Bulletin* **2018**, *98*, 173-179. DOI: https://doi.org/10.1016/j.materresbull.2017.10.007.

(12) Mahana, S.; Manju, U.; Topwal, D. Giant magnetocaloric effect in $GdAlO_3$ and a comparative study with $GdMnO_3$. *Journal of Physics D: Applied Physics* **2017**, *50* (3), 035002. DOI: https://doi.org/10.1088/1361-6463/50/3/035002.

(13) Das, M.; Roy, S.; Mandal, P. Giant reversible magnetocaloric effect in a multiferroic $GdFeO_3$ single crystal. *Phys Rev B* **2017**, *96* (17), 174405. DOI: 10.1103/PhysRevB.96.174405.

(14) Ramirez, A. P. Strongly Geometrically Frustrated Magnets. *Annual review of materials science* **1994**, *24* (1), 453-480. DOI: https://doi.org/10.1146/annurev.ms.24.080194.002321.

(15) Franco, V.; Blázquez, J. S.; Ingale, B.; Conde, A. The Magnetocaloric Effect and Magnetic Refrigeration Near Room Temperature: Materials and Models. *Annual Review of Materials Research* **2012**, *42* (Volume 42, 2012), 305-342. DOI: https://doi.org/10.1146/annurev-matsci-062910-100356.

(16) Brown, I. D.; Altermatt, D. Bond-valence parameters obtained from a systematic analysis of the Inorganic Crystal Structure Database. *Acta Crystallographica Section B* **1985**, *41* (4), 244-247. DOI: https://doi.org/10.1107/S0108768185002063.

(17) Brown, I. D. *Bond valence parameters*. 2020. https://www.iucr.org/resources/data/datasets/bond-valence-parameters (accessed 26/09/2025).

(18) Zhitomirsky, M. E. Enhanced magnetocaloric effect in frustrated magnets. *Phys Rev B* **2003**, *67* (10), 104421. DOI: https://doi.org/10.1103/PhysRevB.67.104421.

(19) Sosin, S. S.; Prozorova, L. A.; Smirnov, A. I.; Golov, A. I.; Berkutov, I. B.; Petrenko, O. A.; Balakrishnan, G.; Zhitomirsky, M. E. Magnetocaloric effect in pyrochlore antiferromagnet $Gd_2Ti_2O_7$. *Phys Rev B* **2005**, *71* (9), 094413. DOI: https://doi.org/10.1103/PhysRevB.71.094413.

(20) Koskelo, E. C.; Mukherjee, P.; Liu, C.; Sackville Hamilton, A. C.; Ong, H. S.; Castelnovo, C.; Zhitomirsky, M. E.; Dutton, S. E. Comparative Study of Magnetocaloric Properties for Gd^{3+} Compounds with Different Frustrated Lattice Geometries. *PRX Energy* **2023**, *2* (3), 033005. DOI: https://doi.org/10.1103/PRXEnergy.2.033005.
